# Kinome Array Profiling of Patient-Derived Pancreatic Ductal Adenocarcinoma Identifies Differentially Active Protein Tyrosine Kinases

**DOI:** 10.3390/ijms21228679

**Published:** 2020-11-17

**Authors:** Justin F. Creeden, Khaled Alganem, Ali S. Imami, F. Charles Brunicardi, Shi-He Liu, Rammohan Shukla, Tushar Tomar, Faris Naji, Robert E. McCullumsmith

**Affiliations:** 1 Department of Neurosciences, College of Medicine and Life Sciences, University of Toledo, Toledo, OH 43614, USA; khaled.alganem@rockets.utoledo.edu (K.A.); ali.imami@rockets.utoledo.edu (A.S.I.); rammohan.shukla@utoledo.edu (R.S.); robert.mccullumsmith@utoledo.edu (R.E.M.); 2 Department of Cancer Biology, College of Medicine and Life Sciences, University of Toledo, Toledo, OH 43614, USA; francis.brunicardi@utoledo.edu (F.C.B.); shi-he.liu@utoledo.edu (S.-H.L.); 3 Department of Surgery, College of Medicine and Life Sciences, University of Toledo, Toledo, OH 43614, USA; 4 PamGene International BV, 5200 BJ’s-Hertogenbosch, The Netherlands; ttomar@pamgene.com (T.T.); fnaji@pamgene.com (F.N.); 5 Neurosciences Institute, ProMedica, Toledo, OH 43606, USA

**Keywords:** peptide array, pancreatic cancer, fibrosis, desmoplasia, transcription factors, kinase inhibitors, kinase signatures, kinomic networks, cancer metabolism, inflammation

## Abstract

Pancreatic cancer remains one of the most difficult malignancies to treat. Minimal improvements in patient outcomes and persistently abysmal patient survival rates underscore the great need for new treatment strategies. Currently, there is intense interest in therapeutic strategies that target tyrosine protein kinases. Here, we employed kinome arrays and bioinformatic pipelines capable of identifying differentially active protein tyrosine kinases in different patient-derived pancreatic ductal adenocarcinoma (PDAC) cell lines and wild-type pancreatic tissue to investigate the unique kinomic networks of PDAC samples and posit novel target kinases for pancreatic cancer therapy. Consistent with previously described reports, the resultant peptide-based kinome array profiles identified increased protein tyrosine kinase activity in pancreatic cancer for the following kinases: epidermal growth factor receptor (EGFR), fms related receptor tyrosine kinase 4/vascular endothelial growth factor receptor 3 (FLT4/VEGFR-3), insulin receptor (INSR), ephrin receptor A2 (EPHA2), platelet derived growth factor receptor alpha (PDGFRA), SRC proto-oncogene kinase (SRC), and tyrosine kinase non receptor 2 (TNK2). Furthermore, this study identified increased activity for protein tyrosine kinases with limited prior evidence of differential activity in pancreatic cancer. These protein tyrosine kinases include B lymphoid kinase (BLK), Fyn-related kinase (FRK), Lck/Yes-related novel kinase (LYN), FYN proto-oncogene kinase (FYN), lymphocyte cell-specific kinase (LCK), tec protein kinase (TEC), hemopoietic cell kinase (HCK), ABL proto-oncogene 2 kinase (ABL2), discoidin domain receptor 1 kinase (DDR1), and ephrin receptor A8 kinase (EPHA8). Together, these results support the utility of peptide array kinomic analyses in the generation of potential candidate kinases for future pancreatic cancer therapeutic development.

## 1. Introduction

**Figure 1:**
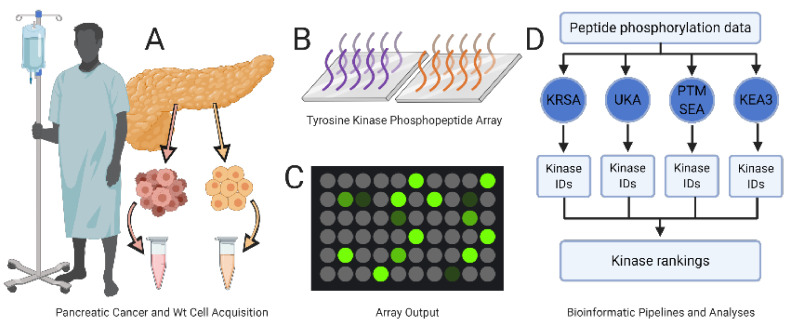
Experimental design. (**A**) Patient-derived pancreatic cancer cells (light red) and wild-type pancreatic tissue specimens (yellow) are processed and diluted to a uniform protein concentration. (**B**) Samples are added to the PamChip array containing 196 consensus phosphopeptide sequences immobilized on porous ceramic membranes; two (purple and orange) such sequences are illustrated here. (**C**) Quantification of peptide phosphorylation levels. (**D**) Peptide phosphorylation data are analyzed with each of four independent bioinformatic pipelines (KRSA, UKA, PTM-SEA, KEA3) and then combined to generate a list of tyrosine protein kinases’ targets.

In vivo, kinases are heavily trafficked and compartmentalized to microdomains and cellular substructures where they act in concert with other kinases, receptors, and effector proteins to realize intricate signaling mechanisms that give rise to complex cellular behaviors. Despite this complexity, kinases are traditionally studied in isolation: A kinase of interest is typically purified from a sample and the enzymatic activity of the isolated kinase is subsequently interrogated. Kinase isolation is often dependent upon antibody binding and subject to the challenges and expenses of antibody-based purification techniques. Traditional assays remove each kinase of interest from its interacting partners and the physiologic conditions in which it normally operates. By breaking these networks into discrete components and examining individual kinases in isolation, researchers discover initiators of kinase phosphorylation activity and catalogue the peptide targets that are activated or deactivated by direct phosphorylation by a specific kinase. Unfortunately, these purification techniques incentivize researchers to minimize the integrity of a kinase’s normal physiologic environment in favor of a clean, easily assayable sample. However, the cleaner a purified kinase sample becomes, the less it can represent that kinase’s activity under physiological endogenous conditions. While this reductionism continues to allow researchers to meaningfully investigate discrete components of kinomic signaling networks, the knowledge gained remains incomplete. In this study, we combined PamGene multiplexed kinome activity array data with four bioinformatic pipelines to identify protein kinases responsible for the differential phosphorylation activity observed in patient-derived and commercial pancreatic ductal adenocarcinoma (PDAC) cell lines compared to patient-derived wild-type pancreatic tissue specimens (Figure 1).

Protein kinases are enzymes capable of phosphorylating other proteins to regulate biochemical signaling pathways and modulate cellular behavior [1]. In cancer, many protein kinases are associated with cancer cell initiation, progression, and metastasis, as well as relapse and survival. Small-molecule protein kinase inhibitors represent an increasingly successful therapeutic strategy for a range of cancer types with over 50 FDA-approved protein kinase inhibitors in clinical use [2,3,4] and hundreds more undergoing clinical or preclinical trials. Protein kinase inhibitors improve patient outcomes significantly and protein kinases are, therefore, a popular target for difficult-to-treat malignancies such as pancreatic cancer. Pancreatic cancer patients demonstrate the lowest five-year relative survival rates of any cancer type, with decades of medical research unable to increase these rates beyond 9% [5]. Protein kinase inhibitors erlotinib (OSI-774; Tarceva), everolimus (RAD001; Afinitor), and sumitinib (SU11248; Sutent) are approved for the treatment of pancreatic cancers [4], though the majority of these approvals are for a rare (1% to 2%) subtype of pancreatic cancer known as pancreatic neuroendocrine tumors [6] rather than the far more common PDAC.

**Figure 2:**
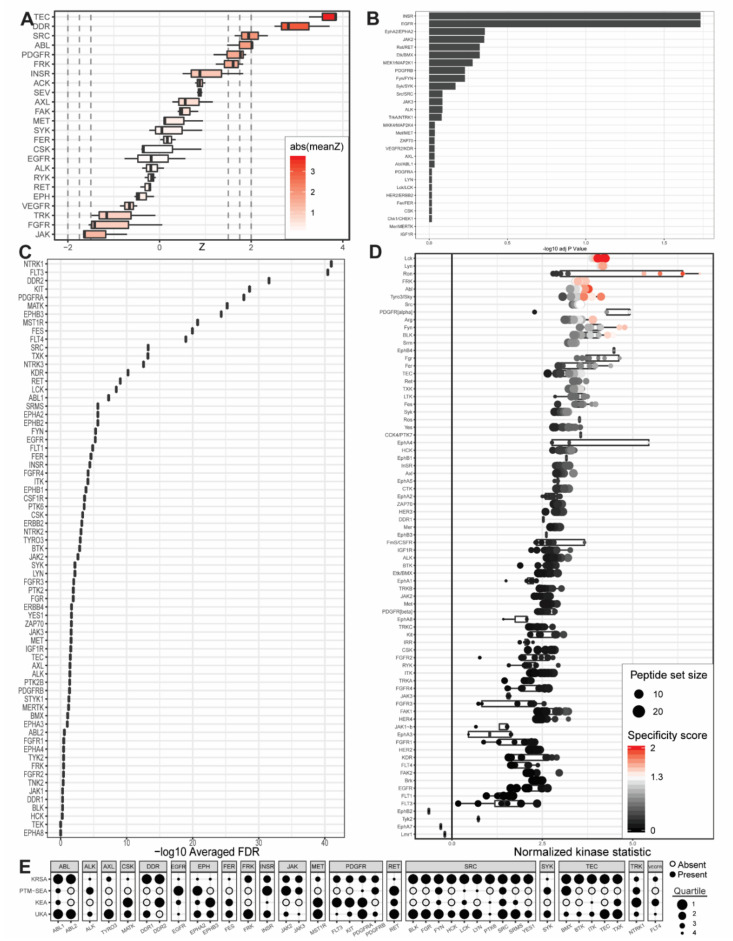
Outputs from upstream kinase identification pipelines for the commercially available PANC1 PDAC cell line compared to patient-derived wild-type pancreatic tissue. (**A**) Kinome Random Sampling Analyzer (KRSA); (**B**) Post-Translational Modification Signature Enrichment Analysis (PTM-SEA); (**C**) Kinase Enrichment Analysis Version 3 (KEA3); (**D**) Upstream Kinase Analysis (UKA); (**E**) Quartile summary. A more detailed figure legend can be found in Appendix B.

**Figure 3:**
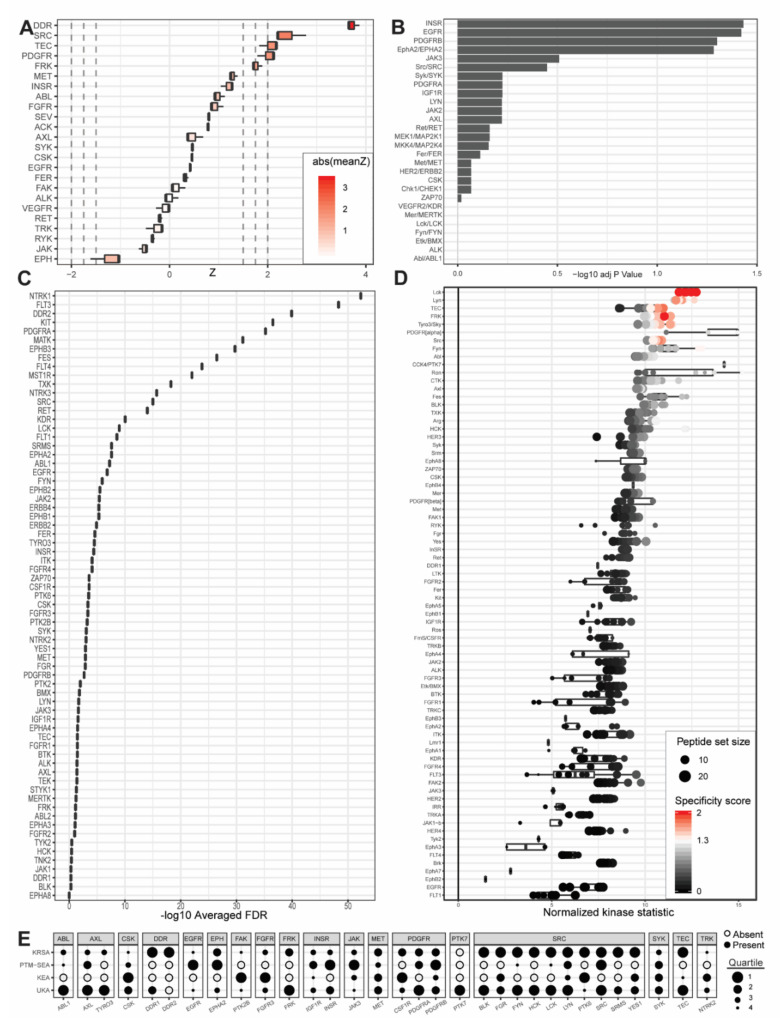
Outputs from upstream kinase identification pipelines for the patient-derived PDCL15 PDAC cell line compared to patient-derived wild-type pancreatic tissue. (**A**) Kinome Random Sampling Analyzer (KRSA); (**B**) Post-Translational Modification Signature Enrichment Analysis (PTM-SEA); (**C**) Kinase Enrichment Analysis Version 3 (KEA3); (**D**) Upstream Kinase Analysis (UKA). (**E**) Quartile summary. A more detailed figure legend can be found in Appendix B.

**Figure 4:**
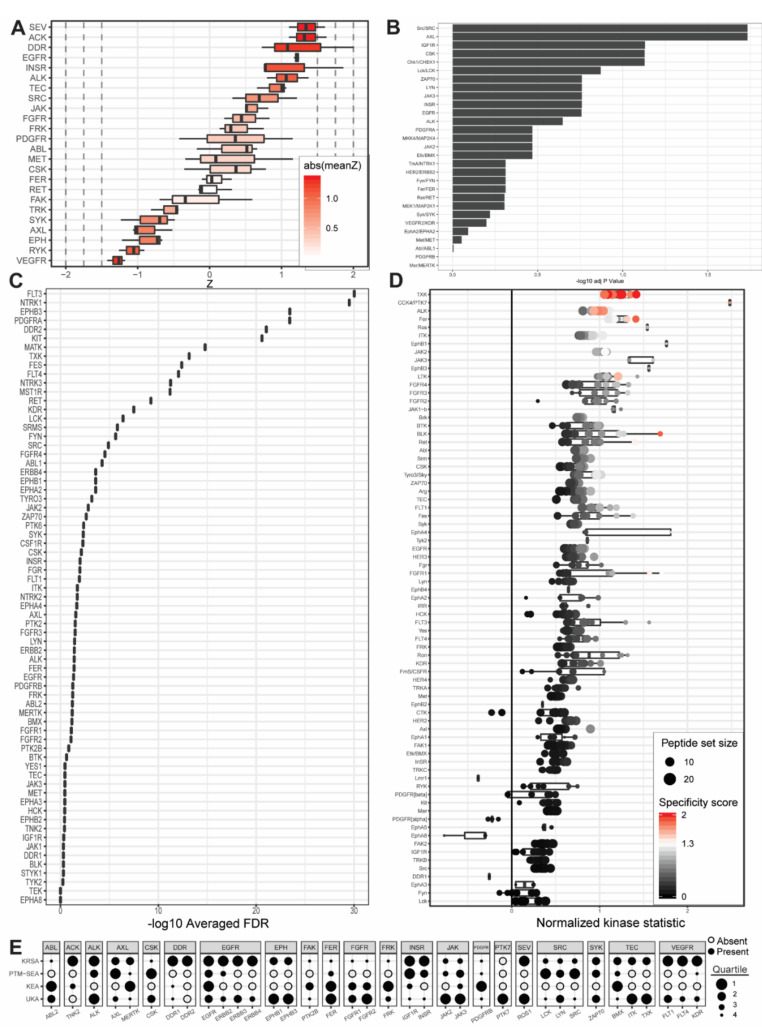
Outputs from upstream kinase identification pipelines for the patient-derived PDCL5 PDAC cell line compared to patient-derived wild-type pancreatic tissue. (**A**) Kinome Random Sampling Analyzer (KRSA); (**B**) Post-Translational Modification Signature Enrichment Analysis (PTM-SEA); (**C**) Kinase Enrichment Analysis Version 3 (KEA3); (**D**) Upstream Kinase Analysis (UKA). (**E**) Quartile summary. A more detailed figure legend can be found in Appendix B.

Previous, excellent genomic and proteomic studies have characterized PDAC subtypes [7,8,9,10,11]. Different and new PDAC subtypes continue to be defined, with myriad genetic or molecular factors increasing the resolution of the disease spectrum. While the present study could not comprehensively address the complexity and heterogeneity of PDAC tumor subtyping, our results inform growing understanding of the unique protein tyrosine kinase networks that play a role in one or more of the commercial and patient-derived cell lines investigated (Figure 2, Figure 3 and Figure 4).

To define kinases that are differentially active in one or more PDAC cell lines and identify potentially actionable drug targets, we sought to break away from linear, one-by-one investigations of individual PDAC kinases and consider instead the totality of PDAC kinase networks within each group. To accomplish this, we combined emerging laboratory technologies and contemporary bioinformatic pipelines to identify lead candidate kinases based on peptide phosphorylation signatures. These technologies include the PamStation and the Protein Tyrosine Kinase PamChip. The bioinformatic pipelines include the Kinome Random Sampling Analyzer (KRSA) pipeline, developed by our own laboratory [12,13,14,15,16,17,18,19], and the Upstream Kinase Analysis (UKA) pipeline, which is part of the BioNavigator software tool developed by collaborators at PamGene [20,21]. To passively validate and further contextualize the results of this approach, two additional bioinformatic pipelines were utilized. These additional bioinformatic pipelines include the Post-Translational Modification Signature Enrichment Analysis (PTM-SEA) pipeline developed at the Broad Institute of MIT and Harvard [22], as well as the Kinase Enrichment Analysis Version 3 (KEA3) developed by the Ma’ayan laboratory [23]. Using laboratory equipment designed by PamGene, we measured the relative phosphorylation levels of 198 representative peptide substrates for PDAC cells and wild-type pancreatic tissue before running the resultant data through each of these four self-contained analytical platforms. The analytical results from each pipeline were interpreted individually as well as in combination to maximize the strengths of each pipeline’s respective algorithm and reference database set. The results were synthesized to identify kinases likely responsible for the unique phosphorylation signatures of PDAC (Figure 2, Figure 3 and Figure 4).

## 2. Results

### 2.1. PamGene Kinome Activity Profiling Using Protein Tyrosine Kinase PamChip^®^

The PamChip is a kind of array. Groups of identical peptide fragments populate a single spot on the chip (Figure 1). The peptide spots contain amino acid sequences of sufficient length and complexity to represent the phosphorylation sites of specific proteins. When these peptide spots are exposed to experimental samples, they become phosphorylated by the activated kinases within that sample. Phosphorylation events correspond precisely with the enzymatic activity of the sample’s kinase networks. Fluorescently tagged phosphoantibodies produce a signal proportional to the phosphorylation activity of the kinases contained within the sample. Using specific collections of peptide fragments, the intensities reported by a PamChip provide a signature that can be used to identify the kinases responsible for the observed phosphorylation activity. UKA or KRSA pipelines process differentially phosphorylated peptide fragment identities and signal intensities. For PTM-SEA and KEA3 pipelines to evaluate PamChip signatures, the peptide fragments must first be converted to protein identities. Because some peptide spots on the PamChip represent multiple proteins and because multiple peptide spots may represent different sites on a single protein, this conversion results in a loss of information. The signal intensities on the PamChip—which quantify the degree to which a peptide fragment has been phosphorylated—are also lost when PamChip signatures are converted to the binary “differentially expressed” input categories required for PTM-SEA or KEA3 pipelines. In other words, instead of interpreting “peptide-fragment-1 with signal intensity 10.3,” PTM-SEA or KEA3 only process “protein-a.” For this reason, we preferentially used the UKA and KRSA pipelines for candidate kinase identification, using the less well-suited (but excellent in their own right) PTM-SEA and KEA3 pipelines for passive validation.

Unabridged comparison of the upstream kinases that each pipeline identified as being responsible for the observed phosphorylation patterns of each pancreatic cancer cell line compared to control wild-type patient-derived pancreatic cells are presented in Figure 2, Figure 3 and Figure 4 as well as Supplementary Table S1. The databases associated with each pipeline offer different levels of kinase coverage. This is advantageous for identification of true positives, although the absence of a kinase from a pipeline’s identification output cannot be considered a negative indication of that kinase serving as a causative factor in the phosphorylation patterns observed. This approach, therefore, minimizes type I error (i.e., erroneous rejection of a true null hypothesis) and accommodates type II error (i.e., erroneous acceptance of a false null hypothesis). UKA and KRSA bioinformatic pipelines are specifically designed to analyze PamChip kinome activity data. As such, each respective algorithm can integrate multiple PamChip data metrics into its final analytical output. The PTM-SEA and KEA3 pipelines, although capable of excellent analytic activity, are designed for slightly different applications. In their native state, significant programmatic alterations are required to generate results that meaningfully interpret PamChip experimental data. The most significant differences between UKA or KRSA bioinformatic pipelines and PTM-SEA or KEA3 bioinformatic pipelines relate to how these two groups handle multiple dimensions of data and how these two groups natively interpret phosphorylated peptides.

### 2.2. UKA and KRSA Combinatory Analysis

In Table 1, we average the percentile rankings without any concern for false negatives. The goal was to increase positive identification of kinases whose activity is truly a causative factor in the differential phosphorylation patterns observed between tumor and wild-type cells. In Table 2, we attempt to decrease the presentation of false positives by dividing these averages by the number of pipelines employed—in this case, two. In Table 1 and Table 2, we also combine the results from the two patient-derived pancreatic cancer cell lines (PDCL15, PDCL5; Patient-Derived) and we combine the results of all pancreatic cancer cell lines (PDCL15, PDCL5, PANC1; All), in order to generate average percentile rankings that inform the general kinase activity of pancreatic cancer. These tables contain only the highest scoring kinases according to UKA and KRSA bioinformatic pipelines. If two or more kinases received identical average percentile ranks or identical weighted average percentile ranks, then rank order was determined arbitrarily. While our complete output data may be found in Table S1, the present tables include only the top 10 highest ranked kinases. Kinases with identical scores were arbitrarily assigned sequential ranks.

### 2.3. Expanded PTM-SEA and KEA3 Combinatory Analysis

Table 3 and Table 4 expand our analyses to include results obtained through PTM-SEA and KEA3 bioinformatic pipelines. Table 3 provides unweighted average percentile rankings. Table 4 attempts to decrease false positives by weighting these averages according to the number of pipelines which identify a given kinase as being responsible for the observed phosphorylation differences between a pancreatic cancer cell line and patient-derived wild-type pancreas. As above, Table 3 and Table 4 also combine results from our two patient-derived pancreatic cancer cell lines (PDCL15, PDCL5; Patient-Derived) as well as from all pancreatic cancer cell lines (PDCL15, PDCL5, PANC1; All).

## 3. Discussion

### 3.1. Identification of Lead Candidate Kinases

Our results confirm the activity of known protein tyrosine kinase-related pathways previously reported as perturbed in pancreatic cancer [24,25,26,27,28]. These results also identify protein tyrosine kinases as yet understudied or unreported in pancreatic cancer. Because our experimental model allows us to maintain the integrity of kinase networks, our data suggest involvement of signaling pathways and regulatory cascades in pancreatic cancer pathophysiology. This study presents evidence in support of the continued development of previously established inhibitory therapeutics that target select protein kinases, such as epidermal growth factor receptor (EGFR) [25], ephrin receptor A2 (EPHA2) [29], and SRC proto-oncogene kinase (SRC) [27], and our experimental results identify new PDAC targets, such as B lymphoid kinase (BLK), lymphocyte cell-specific kinase (LCK), and ABL proto-oncogene 2 kinase (ABL2), which may play a critical role in cancer cell biochemistry or desmoplastic inflammatory cell behavior.

While data gained from reductionist kinase investigations have previously been used to support other complex biochemical studies such as mass spectrometry-based proteomic studies [30], peptide-based kinome array profiling offers unique advantages. Traditionally, peptides identified as kinase targets are probed in vitro and in vivo to examine the behavior of a given kinase. Following that, genetic techniques that knockdown or constitutively express a given kinase further probe the activity of that kinase within normal or experimental biological milieus. But many of these strategies are unable to measure multiple kinases simultaneously while also maintaining kinomic network integrity in complex biological samples. Peptide-based kinome array profiling can accomplish this by overcoming kinase isolation requirements in order to evaluate hundreds of kinases simultaneously. This study was designed as a series of hypotheses generating experiments. Although our experimental designs did not allow us to draw definitive conclusions, our experimental results fall into one of two major categories, which we have termed “reference kinases” and “neoteric kinases.” The first category, “reference kinases,” represents kinases with well-established roles in human cancer pathophysiology. The second category, “neoteric kinases,” represents candidate kinases potentially contributing to PDAC pathology in new or previously understudied ways.

### 3.2. Reference Kinases

Reference kinases include protein tyrosine kinases identified by our study and subsequent bioinformatic analyses that recapitulate previously reported findings in the field of human cancer biology. Kinases in this category provide reference data that passively validate our experimental observations and contextualize our results within the scope of verified kinase discoveries.

**Figure A1:**
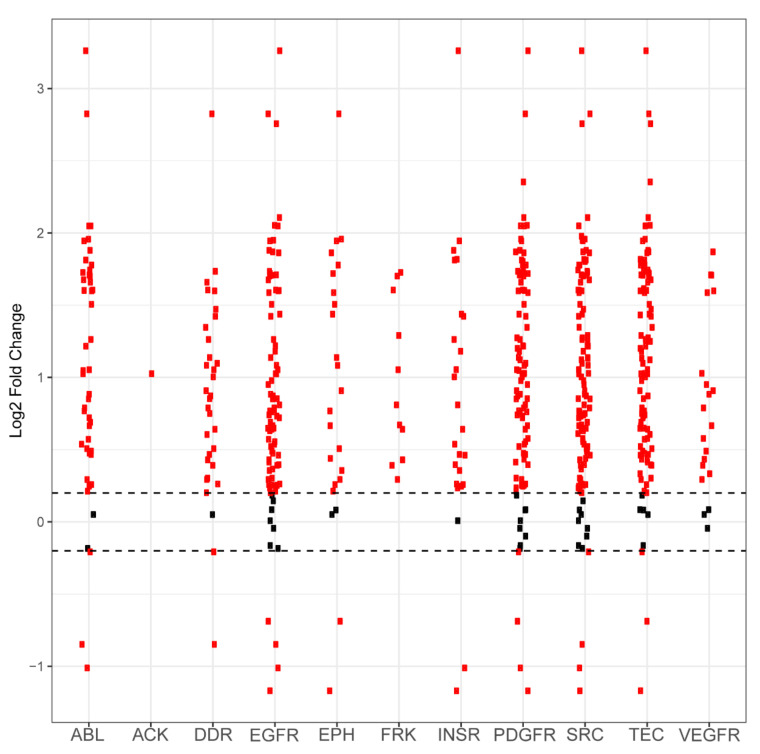
Differential phosphorylation levels of peptide sequences attributed to kinase family activity in PANC1 vs. wild-type control. Each red dot in a column represents one peptide sequence whose phosphorylation is performed by that column’s kinase family. The *y* axis reports log2-fold change and the *x* axis identifies the kinase family. Black dots represent peptides that did not demonstrate differential phosphorylation in PANC1 samples compared to wild-type samples, with the dashed horizontal line representing a positive or negative 0.2 log2-fold change cutoff. Red dots above these lines represent peptides that are more phosphorylated in PANC1 compared to control. Red dots that are below these lines represent peptides that are less phosphorylated in PANC1 compared to control.

**Figure A2:**
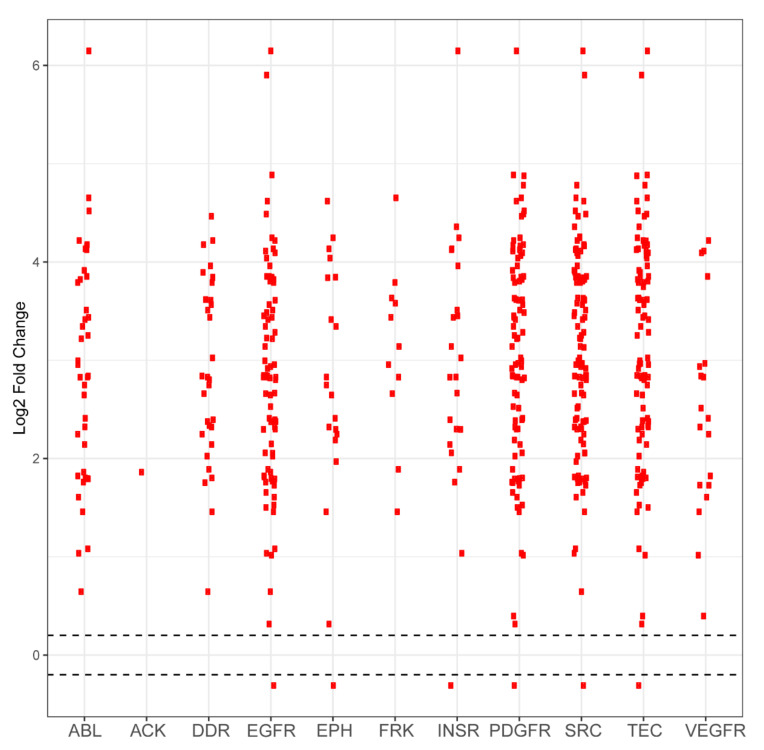
Differential phosphorylation levels of peptide sequences attributed to kinase family activity in PDCL15 vs. wild-type control. Each red dot in a column represents one peptide sequence whose phosphorylation is performed by that column’s kinase family. The *y* axis reports log2-fold change and the *x* axis identifies the kinase family. Black dots represent peptides that did not demonstrate differential phosphorylation in PDCL15 samples compared to wild-type samples, with the dashed horizontal line representing a positive or negative 0.2 log2-fold change cutoff. Red dots above these lines represent peptides that are more phosphorylated in PDCL15 compared to control. Red dots that are below these lines represent peptides that are less phosphorylated in PDCL15 compared to control.

**Figure A3:**
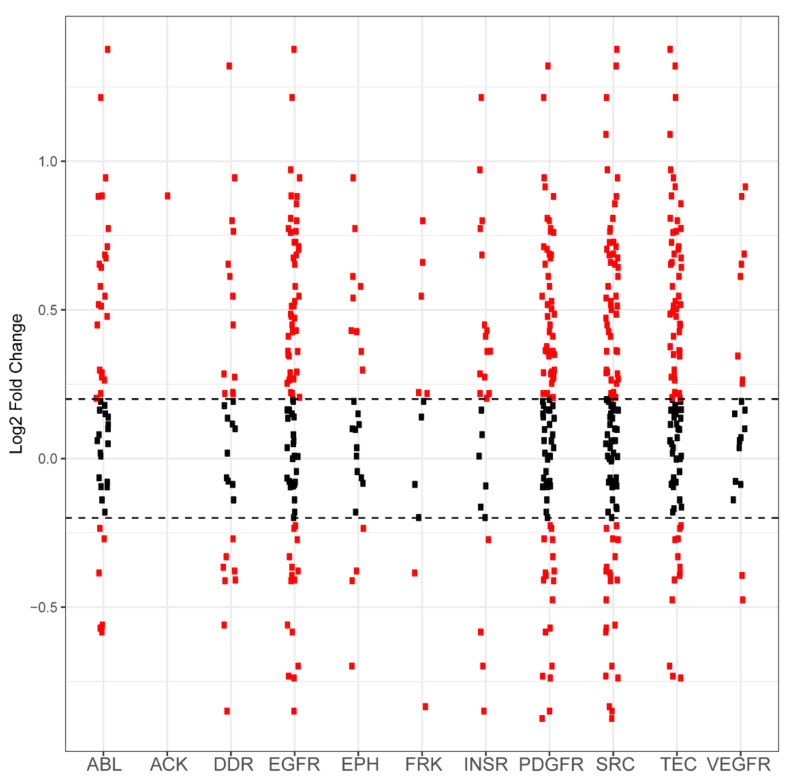
Differential phosphorylation levels of peptide sequences attributed to kinase family activity in PDCL5 vs. wild-type control. Each red dot in a column represents one peptide sequence whose phosphorylation is performed by that column’s kinase family. The *y* axis reports log2-fold change and the *x* axis identifies the kinase family. Black dots represent peptides that did not demonstrate differential phosphorylation in PDCL5 samples compared to wild-type samples, with the dashed horizontal line representing a positive or negative 0.2 log2-fold change cutoff. Red dots above these lines represent peptides that are more phosphorylated in PDCL5 compared to control. Red dots that are below these lines represent peptides that are less phosphorylated in PDCL5 compared to control.

One notable reference kinase identified as differentially active in our study of PDAC cells is the EGFR tyrosine kinase. Previously, EGFR has been linked to pancreatic tumor size, advanced clinical staging, and poor survival [28]. The expression frequency of EGFR in human pancreatic carcinomas is reported as 43% [28] and 68.4% in primary invasive ductal carcinoma of the pancreas [31] with elevated expression of EGFR activating ligands also reported. Consistent with these reports, our results show differential EGFR activity in weighted analyses of PDCL5 (Figure 4, Table 2 and Table 4) and aggregated patient-derived cell lines (Table 4). Directionality (increased kinase phosphorylation activity or decreased kinase phosphorylation activity) within each cell line can be gleaned from KRSA’s report of the log2-fold change of phosphorylated peptide substrates attributed to each kinase family (Figure A1, Figure A2, or Figure A3) or by UKA’s report of an individual kinase’s mean kinase statistic (Table A1). In our study, EGFR demonstrated increased phosphorylation activity in pancreatic cancer compared to control. Inhibition of the EGFR tyrosine kinase improves survival in PDAC animal models [25]. As such, targeted inhibition of tyrosine kinases, including EGFR, is a popular goal of many emerging therapeutic strategies [2]. Erlotinib (OSI-774) is an FDA-approved small-molecule EGFR tyrosine kinase inhibitor for use in pancreatic cancer [3]. Many additional small-molecule tyrosine kinase inhibitors are currently under study or in various stages of clinical trial for their putative role in pancreatic cancer pathophysiology.

The vascular endothelial growth factor receptor (VEGFR) tyrosine kinase family is also heavily implicated in the development of pancreatic cancer [32]. VEGFR-3, also known as fms related receptor tyrosine kinase 4 (FLT4), has been explored as a target for pancreatic cancer therapy [24,33] with significantly upregulated FLT4 expression documented in pancreatic cancer specimens [34,35]. Beyond PDAC, single nucleotide polymorphisms (SNPs) of FLT4 correlate with decreased progression-free survival of patients with gastroenteropancreatic neuroendocrine neoplasms [36]. Our results demonstrate increased FLT4 kinase activity in PDAC cells. In the present study, we identified FLT4 as one of the most differentially active kinases in PDCL5 patient-derived PDAC samples according to average percentile rankings across all pipelines (Table 3). KRSA and UKA directionality metrics (Figure A3, Table A1) demonstrate increased FLT4 activity in pancreatic cancer compared to wild-type controls.

Several kinases consistently identified as differentially active across multiple pipelines, cell lines, or final combinatorial analyses recapitulate kinases previously identified as playing well-established roles in a variety of human cancer pathologies. These kinases include insulin receptor (INSR) kinase [37,38] (Figure 2 and Figure 3, Table 3 and Table 4), EPHA2 kinase [29,39,40,41,42,43,44,45,46] (Figure 2 and Figure 3, Table 4), platelet-derived growth factor receptor alpha (PDGFRA) kinase [47,48,49,50,51] (Figure 2 and Figure 3, Table 1, Table 2, Table 3 and Table 4), SRC kinase [26,27,52,53,54,55,56,57] (Figure 2 and Figure 3, Table 1, Table 2, Table 3 and Table 4), and tyrosine kinase nonreceptor 2 (TNK2) kinase [58,59,60,61,62,63,64] (Figure 4, Table 1). Of these, EPHA2 is particularly well characterized in PDAC with other groups recently presenting evidence of EPHA2-mediated drug resistance in pancreatic cancer cells [29] and proposing EPHA2 as a potential biomarker or therapeutic target in pancreatic cancer [29,40]. SRC, too, has a well-established evidence base supporting its role in PDAC [27]. Furthermore, many of these kinases are known to constitute important signaling axes in pancreatic cancer. PDGFR/SRC signaling is a therapeutic target in pancreatic cancer [26] with reports of SRC also potentiating PDGFRA activity in other cancer models. TNK2 (also known as ACK1) associates with EGFR in cancer cells to maintain EGFR on the cell surface and enhance human breast cancer cell migration and invasion [65]. Again, this relationship seems to have some degree of bidirectionality, with EGFR influencing TNK2 activation [66,67]. Identification of multiple kinase pairs constituting previously reported signaling axes is encouraging and supports the validity of our experimental design in maintaining the integrity of kinomic signaling networks.

These results lend strength not only to our experimental and bioinformatic identification of differentially active kinases in pancreatic cancer, but also suggest that kinases identified as among the most strongly differential (e.g., in the top 10) in unweighted average percentile rankings (Table 1 or Table 3) and in the corresponding weighted average percentile rankings (Table 2 or Table 4) may represent kinases highly likely to contribute to the pathophysiologic processes of PDAC.

### 3.3. Neoteric Kinases

Neoteric kinases represent a second, smaller category of experimental findings that include kinases whose identification in our study and bioinformatic analyses suggest new, hitherto unidentified, or otherwise understudied kinase functionalities in PDAC. Because these kinases are not strictly “novel,” we instead call this group “neoteric” in reference to the emerging roles these kinases may play in pancreatic tumor desmoplasia, immune response, and oncometabolism. Beyond the passive validation that our “reference kinase” group provides, this group of “neoteric kinases” provides potentially novel insights. It became evident, after identifying lead candidate kinases, that our data highlight several potential players in unique aspects of PDAC tumor development. Kinases that appear both in our final unweighted average percentile rankings and in our final weighted average percentile rankings are defined as lead candidate kinases and include BLK (Figure 2 and Figure 3, Table 1 and Table 2), Fyn-related kinase (FRK) (Figure 3, Table 1 and Table 2), Lck/Yes-related novel kinase (LYN) (Figure 2 and Figure 3, Table 1, Table 2, and Table 4), FYN proto-oncogene kinase (FYN) (Figure 2 and Figure 3, Table 1, Table 2, Table 3 and Table 4), LCK (Figure 2 and Figure 3, Table 1, Table 2, Table 3 and Table 4), and tec protein kinase (TEC) (Figure 2 and Figure 3, Table 1, Table 2 and Table 3). Additional kinases identified by one or more bioinformatic pipelines define candidate kinases and include hemopoietic cell kinase (HCK) (Figure 3, Table 2), ABL2 (Figure 2, Table 2), discoidin domain receptor 1 kinase (DDR1) (Table S1), and ephrin receptor A8 kinase (EPHA8) (Table S1). While some of these kinases have been previously identified in kinome or phosphorylome studies of pancreatic cancer (e.g., DDR1 [9], FYN [68]), we classified them as neoteric for the purposes of this discussion because sufficient questions remain as to how these kinases relate to PDAC pathology, treatment, or molecular signaling.

Pronounced deposition of extracellular matrix constituents and the aberrant propagation of fibroblasts characteristic of desmoplasia are common in PDAC tumor microenvironments. Desmoplasia acts as a biophysical barrier contributing to pancreatic cancer therapeutic resistance. Desmoplastic stroma and pancreatic tumor cells interact with one another to elicit complex cellular behaviors with seemingly contradictory roles in PDAC progression [69]. At times pro-tumorigenic [70] and at times anti-tumoral [69], the role of desmoplasia in PDAC remains an active area of study. Our identification of increased HCK, ABL2, DDR1, FYN, and LYN suggests a role for these kinases in the desmoplastic reactions that contribute to the poor survival rates of PDAC patients. HCK overexpression activates fibrotic pathways [71]. ABL2 signaling regulates fibroblast proliferation [72]. DDR1 inhibitors reduce fibrosis in other fibrotic diseases [73,74,75,76]. FYN regulates downstream serine-threonine kinase activities involved in the modulation of fibroblast–epithelial cell interactions and the promotion of organ fibrosis [77,78]. Serotonin promotes fibroblast activation and collagen deposition [79]. LYN mediates pro-tumor serotonin signaling [80].

Desmoplasia serves as the primary source of the cytokines and chemokines that facilitate tumor progression in PDAC [81]. While immunotherapeutic strategies have significantly impacted clinical success in many other human malignancies, pancreatic cancer remains resistant. LCK is an important regulator of immune cell functionality [82]. TEC is a key player in the inflammatory response of pancreatitis [83]. The LCK and TEC kinases identified in the present study may also play a role in the anti-cancer immune response elicited and frequently evaded by PDAC.

The final effector proteins and terminal nodes for many kinase cascades are transcription factors. Our identification of BLK as a differentially active tyrosine kinase in PDAC cells compared to wild-type pancreatic cells presents new insight into the role that the pancreatic and duodenal homeobox 1 (PDX1) transcription factor plays in tumor progression. Overexpression of BLK induces an increase in the PDX1 transcription factor in the cytoplasm and nucleus [84]. The biological functionalities of the PDX1 transcription factor are multitudinous and context dependent. Our group has published extensively on the pro-tumorigenic role of the PDX1 transcription factor in pancreatic cancer progression [85,86,87,88,89,90,91], while other groups have demonstrated antimetastatic [92] and tumor-suppressive effects [93]. Recent evidence shows PDX1 functionality and its multiple—often antagonistic—effects on pancreatic cancer are stage-specific [93,94]. Our data suggest potential mechanistic relationships by which BLK kinase signaling cascades may contribute to PDX1′s multifaceted role in PDAC.

PDX1, originally known as insulin promoter factor 1 (IPF1), also serves as a transcriptional activator for metabolic genes such as insulin and glucose transporter type 2. Intersection between the PDX1 transcription factor, the BLK, and INSR kinases, as well as the role these kinases play in oncometabolic processes, will be examined in future studies. PDAC cells experience extreme deprivation of nutrient and oxygen delivery. Our data also implicate LYN, EPHA8, and FYN kinases as potential actors in oncometabolic PDAC signaling pathways and suggest mechanisms by which these kinases may facilitate oncogenic behavior.

**Figure A4:**
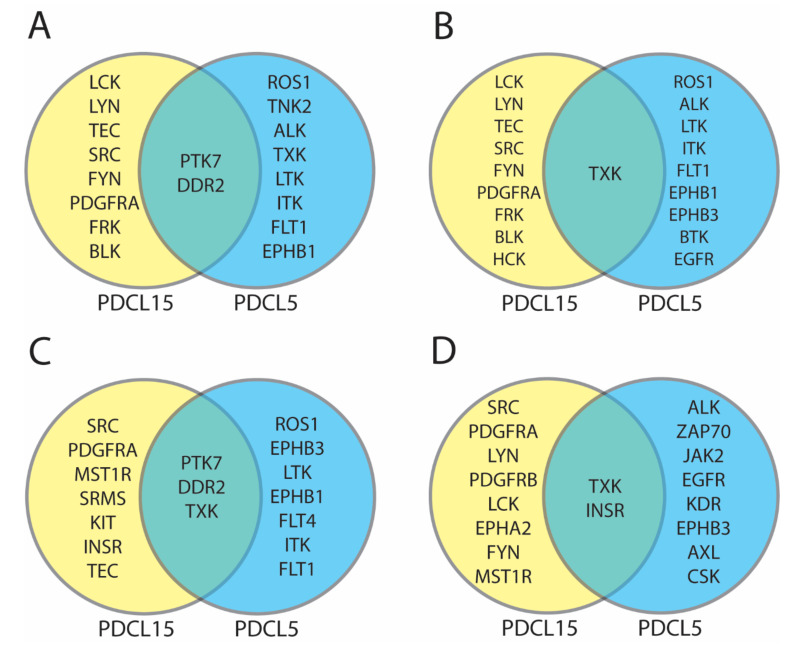
Comparison of protein tyrosine kinases identified in patient-derived cell lines. For each comparison yellow circles represent differentially active protein tyrosine kinases in PDCL15, blue circles represent differentially active protein tyrosine kinases in PDCL5, and green overlapping area represents differentially active protein tyrosine kinases in PDCL15 and PDCL5. (**A**) Comparison of top 10 differentially active protein tyrosine kinases according to UKA and KRSA average percentile rankings. (**B**) Comparison of top 10 differentially active protein tyrosine kinases according to UKA and KRSA weighted average percentile rankings. (**C**) Comparison of top 10 differentially active protein tyrosine kinases according to all pipelines (KRSA, UKA, PTM-SEA, and KEA3) average percentile rankings. (**D**) Comparison of top 10 differentially active protein tyrosine kinases according to all pipelines (KRSA, UKA, PTM-SEA, and KEA3) weighted average percentile rankings.

Identification of these kinases in the present study contributes to growing understanding of abnormal fibrotic processes prominent in PDAC, dysregulated transcription factor activity, anti-cancer immune response, and the complex kinomic signaling networks responsible for pathometabolic tumor regulation and nutrient delivery. As novel PDAC subtypes continue to be defined [7,8,10,11,95,96,97], it is clear that therapeutic strategies for PDAC must take different genetic backgrounds into account. The mutational profiles for the patient-derived cell lines used in this study (Table A2) provide useful insights into which protein tyrosine kinases may serve as effective targets of personalized/precision therapeutic intervention (Figure A4).

## 4. Materials and Methods

### 4.1. Experimental Design

The experimental design is illustrated in Figure 1. In brief, PDAC epithelial cells and normal pancreatic ductal epithelial cells derived from patients were subjected to kinome array analysis using the PamStation 12 platform. All samples were prepared and assayed sequentially using Tyrosine Kinase PamChips consisting of 196 peptides with known phosphorylation sequences representing over 100 different proteins associated with the activity of upstream kinases. Each sample was assayed in triplicate with the results averaged across three identical kinome array runs.

### 4.2. Cell Lines and Patient-Derived Tissue

We used three different pancreatic cancer cell lines: one commercial cell line and two patient-derived cell lines (PDCL). We used commercial (ATCC CRL-1469) PANC1 cells originating from human pancreatic ductal cells carrying a *TP53_R273H* mutation and a *KRAS_G12D* mutation [98]. Two patient-derived cell lines (PDCL5, original name TKCC-05; PDCL-15, original name TKCC-15-Lo) were kindly provided by Andrew Biankin from Wolfson Wohl Cancer Research Centre, UK, with authentication by STR [90]. PDCL5 carries a *TP53_G245S* mutation and a *KRAS_G12V* mutation, while PDCL15 carries only a *KRAS_G12D* mutation (Table A2). These mutational profiles were kindly provided by Andrew Biankin and confirmed by F. Charles Brunicardi and Shi-He Liu. Normal patient-derived pancreatic ductal tissue was harvested from a healthy donor and kindly provided by Camillo Ricordi at the Diabetes Research Institute, University of Miami Miller School of Medicine, under the material transfer agreement. Because traditional two-dimensional cell culture models fail to accurately represent cancer microenvironments, we endeavored to obtain control tissue that more accurately represents physiological conditions. The pancreatic tissue contains ductal cells, acinar cells, and other elements included in the pancreatic microenvironment. While our decision to compare cell lines with wild-type pancreatic tissue may introduce a degree of bias into the study (cell lines and tissue samples contain different cellular contexts and environments), we used identical control wild-type tissue for each cell line comparison. All experiments and procedures were performed in strict compliance with all relevant laws and institutional guidelines.

Cell lines were cultured and lysed 72 h after plating, and tissue samples were processed as previously described [90]. All procedures were performed on ice. Tissue homogenization was performed using a D2400 Homogenizer and 1.5-mm Triple-Pure Zirconium Beads, with five rounds of homogenization and liquid nitrogen cooling to maintain low temperatures and minimize protein degradation. Each round of homogenization consisted of three cycles, with each cycle consisting of 30 s of active homogenization at 7 m/s and 30-second intervals between cycles. Tissue and cell lysate protein extractions were performed using M-PER (mammalian protein extraction reagent) (ThermoFisher, Waltham, MA, USA) and Halt Protease and Phosphatase Inhibitor Cocktails (ThermoFisher). Samples were centrifuged (14,000 RPM, 10 min, 4 °C) before supernatant collection. Total protein concentrations were assayed (Pierce BCA Protein Assay Kit, ThermoFisher) and samples were diluted to 1 µg/µL. All samples were prepared and measured simultaneously. Because freeze–thaw cycles decrease kinase activity [99], multiple aliquots were stored at −80 °C to minimize freeze–thaw cycles, with frozen aliquots used only once for kinome array assays.

### 4.3. Tyrosine Kinase Array

Tyrosine kinase activity was measured with the PamStation 12 instrument (PamGene International, ’s-Hertogenbosch, The Netherlands) and PTK (4-well) array PamChips using fluorescently labeled antibodies to detect differential phosphorylation of 196 reporter peptides (including three internal controls) per well. These 196 consensus phosphopeptide sequences were immobilized on porous ceramic membranes. The PamChip wells were blocked with 2% bovine serum albumin (BSA) prior to addition of 1 µg of protein suspended in manufacturer’s kinase buffer (PamGene). Next, we added 157 µM adenosine triphosphate (ATP) and FITC-labeled anti-phospho tyrosine antibodies (PamGene) to each well. Homogenized lysates containing active kinases and assay solution were pumped back and forth through PamChip wells in order to facilitate interactions between the active kinases and the 196 immobilized consensus phosphopeptide sequences. Evolve (PamGene) software captured FITC-labeled anti-phospho-antibodies bound to the phosphorylated consensus sequences. Image capture occurred every six seconds for 60 min. After washing, peptide signal intensity was recorded across several exposure times (10, 20, 50, 100, 200 milliseconds). The linear regression slope was calculated in order to provide the peptide phosphorylation intensity signal used in downstream comparative analyses. Signal ratios between pairs of samples were used to calculate fold change (FC) for each peptide. Differential peptide signals greater than or equal to 30% (FC ≥ 1.30 or FC ≤ 0.70) were considered demonstrative of minimum threshold changes in the degree of phosphorylation. This threshold value derived from conservative interpretation of previous literature suggesting even smaller orders of magnitude are sufficiently correlated with biologically relevant signaling changes [16,100,101]. Nonlinear (R^2^ values less than 0.90) or undetectable peptides in the post-wash phase were not selected for further analysis. Kinome assays were performed in triplicate with the calculated FC per peptide averaged across three replicates.

### 4.4. Upstream Kinase Identification

Kinase activity corresponded to the degree of consensus peptide phosphorylation as measured using real-time Evolve kinetic image capture software. The raw data generated by the PamStation platform were minimally processed (using threshold changes described above) to generate a list of differentially phosphorylated peptide sequences, which served as input for subsequent bioinformatic analyses. To expand coverage of peptide sequences and maximize identification of candidate upstream kinases, we used four distinct bioinformatic pipelines, each with a semi-overlapping set of databases by which their respective algorithms queried our list of differentially phosphorylated peptide sequences. Because each pipeline also relied upon a unique pipeline-specific algorithm, evaluating the results of one pipeline within the context of the results obtained through the other three pipelines allowed for enhanced perspective. Shared identification of upstream kinases responsible for the observed peptide phosphorylation patterns between pipelines could, therefore, be weighted and integrated into the final analysis. These pipelines include (1) Upstream Kinase Analysis (UKA) from PamGene, (2) Post-Translational Modification Signature Enrichment Analysis (PTM-SEA) from the Broad Institute of MIT and Harvard, (3) Kinase Enrichment Analysis Version 3 (KEA3) from the Ma’ayan laboratory, and (4) Kinome Random Sampling Analyzer (KRSA) developed by our own laboratory [12,13,14,15,16,17]. Meaningful differences between pipelines were compared in the Discussion section, while the methods by which we deployed each pipeline are presented below.

#### 4.4.1. Upstream Kinase Analysis (UKA) Pipeline

Using PamGene’s BioNavigator software, we ran our data sets through the Protein Tyrosine Kinase (PTK) Upstream Kinase Analysis (UKA) Knowledge Integration PamApp. These data sets included (1) mean phosphorylated peptide sequences of PDCL15 vs. wild-type, (2) mean phosphorylated peptide sequences of PDCL5 vs. wild-type, and (3) mean phosphorylated peptide sequences of PANC1 vs. wild-type. Sample names served as the factor uniquely defining each observation. The “treatment off chip” factor served as the factor defining the experimental groupings. The default scan rank (4 to 12) and permutation (500) parameters were applied to the analysis, in addition to the default in vitro/in vivo (1), in silico (PhosphoNET) (1), minimal sequence homology (0.9), minimal PhosphoNET prediction score (300), and minimal peptide set (3) parameters. Inclusive percentile ranks were calculated according to the absolute value of UKA’s Median Final Score output.

#### 4.4.2. Post-Translational Modification Signature Enrichment Analysis (PTM-SEA) Pipeline

We used the Broad Institute’s Single sample Gene Set Enrichment Analysis (ssGSEA) and Post-Translational Modification Signature Enrichment Analysis (PTM-SEA) publicly available repository (https://github.com/broadinstitute/ssGSEA2.0), RStudio Desktop 1.2.5042 (https://rstudio.com), and underlaying R 3.3.1 software environment (https://cran.rstudio.com). We ran our data sets through the PTM-SEA pipeline after modifying the peptide database to include only the peptide sequences present on the PamChip plus all peptide sequences with minimal sequence homologies of 0.9. These data sets include (1) mean phosphorylated peptide sequences of PDCL15 vs. wild-type, (2) mean phosphorylated peptide sequences of PDCL5 vs. wild-type, and (3) mean phosphorylated peptide sequences of PANC1 vs. wild-type. Results were filtered to include only protein tyrosine kinases. PTM-SEA inclusive percentile ranks were determined according to each kinase’s respective 1/fdr.pvalue.totalGeoMeanLFC output value.

#### 4.4.3. Kinase Enrichment Analysis Version 3 (KEA3) Pipeline

We used the Ma’ayan laboratory’s Kinase Enrichment Analysis Version 3 (KEA3) (https://amp.pharm.mssm.edu/kea3/#) to process our data sets. These data sets include (1) mean phosphorylated peptide sequences of PDCL15 vs. wild-type, (2) mean phosphorylated peptide sequences of PDCL5 vs. wild-type, and (3) mean phosphorylated peptide sequences of PANC1 vs. wild-type. To accommodate the input parameters of KEA3, these peptide sequences were converted to HGNC-approved gene symbols before the data sets were entered into the KEA3 pipeline. Results were filtered to include only protein tyrosine kinases. Average FDR p-values from 0.2, 0.3, and 0.4 LFC cutoff input lists were averaged according to ChengKSIN, PTMsigDB, or PhosDAll database outputs. The resultant ChengKSIN, PTMsigDB, and PhosDAll average values were themselves averaged and −log10 transformations of these averages were used for inclusive percentile ranking calculations.

#### 4.4.4. Kinome Random Sampling Analyzer (KRSA) Pipeline

Our laboratory developed Kinome Random Sampling Analyzer (KRSA) (version 2.0, Toledo, OH, USA) to associate differentially phosphorylated peptide sequences with specific kinases [12,13,14,15,16,17]. To accomplish this, we mapped phosphorylation sites within the reporter peptides to individual protein kinases that phosphorylate these sites. To this end, multiple databases were queried including GPS 5.0 (http://gps.biocuckoo.cn), Kinexus Phosphonet (http://www.kinexus.ca), PhosphoELM (http://phospho.elm.eu.org), and PhosphoSite Plus (https://www.phosphosite.org). In this way, peptide sequences and kinases were matched such that ranked predictions were generated to identify tyrosine kinases most likely to have produced the observed phosphorylation results. Kinases with scores greater than twice the prediction threshold for each phosphorylation site in the GPS 5.0 database were carried forward for downstream analysis. The top five kinase predictions in the Kinexus database were also carried forward for downstream analysis. Additional kinases reported to act on specific phosphorylation sites were identified using PhosphoELM and Phosphosite Plus public databases and carried forward for downstream analysis. Downstream analysis consists of 3000 random sampling iterations in which an equal number of differentially phosphorylated peptide sequences are randomly selected from the total list of 196 phosphopeptide sites on the tyrosine kinase PamChip. Predicted kinases were then mapped to each iteration in order to generate comparative controls to which the experimentally generated kinase lists could be compared. This allowed meaningful approximations of the direction of activity (increased or decreased) and significance for each experimentally identified kinase. Inclusive percentile ranks were calculated according to mean LFC output. KRSA is publicly available at https://github.com/kalganem/KRSA. Because KRSA identifies kinase families rather than individual kinases, all kinases within a family were given identical scores.

### 4.5. Combinatory Analyses

To resolve different output metrics, the results of each respective pipeline were converted to inclusive percentile rankings. These inclusive percentile rankings were aggregated according to cell line and protein tyrosine kinase and averaged per cell line (PANC1, PDCL15, PDCL5) or per cell line group (Patient-derived cell line group: PDCL15 and PDCL5; All group: PANC1, PDCL15, PDCL5). Weighted averages were calculated by dividing average percentile rankings by the number of pipelines under consideration.

### 4.6. Peptide Identities, Gene Synonyms, Family Designations, and Other Mapped Data

To resolve different output terms and provide additional contextual information, several sources were consulted for peptide identities, gene synonyms, kinase family designations, and other categorical or descriptive terms. These sources include UniProt’s Human and mouse protein kinases: classification and index (https://www.uniprot.org) [102,103,104], as well as kinase.com (http://kinase.com/) [103], GPS 5.0 (http://gps.biocuckoo.cn) [105,106], The GeneCards’ human gene database (https://www.genecards.org/), and HUGO Gene Nomenclature Committee (HGNC) (https://www.genenames.org/). Full listing of approved human gene nomenclature can be found in Appendix B, Table A3. Nomenclature mapping can be found in Appendix B, Table A4.

### 4.7. Figure Generation

Figures created with BioRender.com (Toronto, Ontario, Canada), Adobe Creative Suite (San Jose, CA, USA), and R (version 3.6.3). Additional figure panels created with KRSA, UKA/BioNavigator, PTM-SEA, or KEA3.

## 5. Conclusions

**Figure 5:**
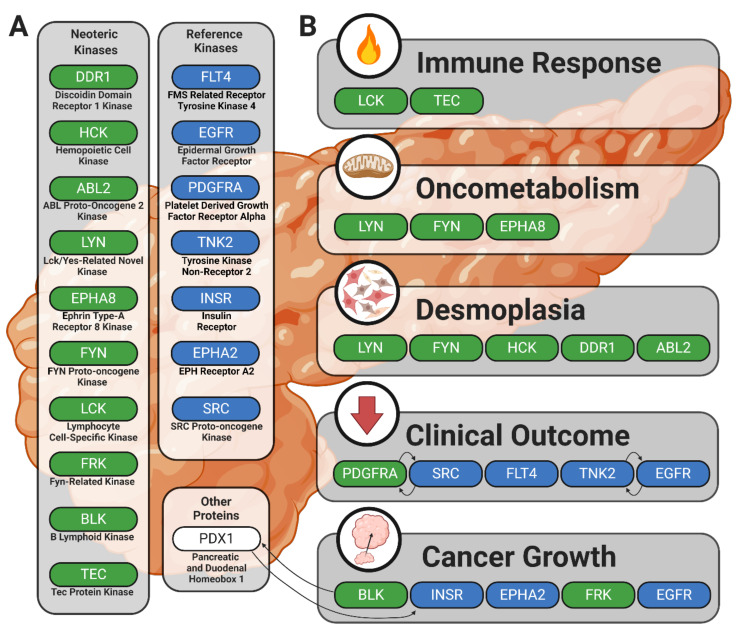
Summary figure illustrating kinases showing increased enzymatic phosphorylation activity in PDAC and their potential roles in the disease process. Solid black arrows indicate relationships between kinases or other proteins. (**A**) The neoteric kinase group includes candidate kinases potentially contributing to PDAC pathology in new or previously understudied ways; the reference kinase group includes kinases with well-established roles in human cancer pathophysiology. (**B**) Kinases are clustered by linkage to the processes that might underlie their involvement in PDAC.

This study provides evidence in support of previously reported kinases in human cancer with an emphasis on PDAC. This passive validation supports the strength of ongoing drug development strategies that target protein tyrosine kinases and propounds the utility and accuracy of peptide-based kinomic analytical platforms. Furthermore, our identification and contextualization of candidate or lead candidate kinases responsible for the differential phosphorylation signatures observed between PDAC commercial or patient-derived cell lines compared to wild-type pancreatic patient samples provides evidence of unique kinomic relationships between pancreatic tumor cells and the desmoplastic stromal environments that support tumor progression and cause significant obstacles in pancreatic cancer therapy. Identification of the BLK, HCK, FRK, ABL2, DDR1, LYN, EPHA8, FYN, LCK, and TEC kinases as potentially significant mediators of pancreatic cancer progression and fibrotic/desmoplastic development fits well into established knowledge while also advancing new avenues of drug development and discovery. Additionally, our data provide increased understanding of the relationship between BLK protein tyrosine kinase, PDX1 transcription factor, and pancreatic disease. This study also outlines additional mechanisms by which HCK, ABL2, and DDR1 may play a role in pancreatic cancer and fibrosis. These results also support the role of LYN in oncometabolic processes and posit pathways by which LYN, EPHA8, and FYN may facilitate oncogenic cellular behavior. Lastly, we provide a rationale for continued investigation of the complex interplay between anti-cancer immune response and the activity of LCK and TEC kinases. These findings are summarized in Figure 5, and our companion review piece provides additional information on the role of BLK, HCK, FRK, ABL2, DDR1, LYN, EPHA8, FYN, LCK, and TEC kinases in PDAC and pancreatic cancer desmoplasia [107].

## Figures and Tables

**Table 1:** UKA and KRSA average percentile rankings.

Cell Line	Rank	Kinase	Family	Average	KRSA	UKA
PANC1	#1	LCK	SRC	96%	91%	100%
PANC1	#2	DDR2	DDR	96%	96%	
PANC1	#3	LYN	SRC	95%	91%	99%
PANC1	#4	SRC	SRC	92%	91%	92%
PANC1	#5	ABL1	ABL	91%	87%	95%
PANC1	#6	TEC	TEC	90%	100%	80%
PANC1	#7	FYN	SRC	90%	91%	88%
PANC1	#8	BLK	SRC	89%	91%	87%
PANC1	#9	TXK	TEC	89%	100%	77%
PANC1	#10	SRMS	SRC	88%	91%	85%
PDCL15	#1	DDR2	DDR	100%	100%	-
PDCL15	#2	LCK	SRC	98%	96%	100%
PDCL15	#3	LYN	SRC	97%	96%	99%
PDCL15	#4	TEC	TEC	94%	91%	97%
PDCL15	#5	SRC	SRC	94%	96%	92%
PDCL15	#6	FYN	SRC	93%	96%	91%
PDCL15	#7	PDGFRA	PDGFR	90%	87%	93%
PDCL15	#8	FRK	FRK	89%	83%	96%
PDCL15	#9	BLK	SRC	88%	96%	81%
PDCL15	#10	PTK7	PTK7	88%	-	88%
PDCL5	#1	PTK7	PTK7	99%	-	99%
PDCL5	#2	ROS1	SEV	97%	100%	95%
PDCL5	#3	TNK2	ACK	96%	96%	-
PDCL5	#4	DDR2	DDR	87%	87%	-
PDCL5	#5	ALK	ALK	86%	74%	97%
PDCL5	#6	TXK	TEC	83%	65%	100%
PDCL5	#7	LTK	ALK	80%	74%	86%
PDCL5	#8	ITK	TEC	79%	65%	93%
PDCL5	#9	FLT1	VEGFR	78%	91%	65%
PDCL5	#10	EPHB1	EPH	76%	61%	92%
Patient-Derived	#1	PTK7	PTK7	100%	-	100%
Patient-Derived	#2	DDR2	DDR	100%	100%	-
Patient-Derived	#3	LYN	SRC	96%	96%	96%
Patient-Derived	#4	TXK	TEC	95%	91%	99%
Patient-Derived	#5	TEC	TEC	94%	91%	97%
Patient-Derived	#6	LCK	SRC	92%	96%	88%
Patient-Derived	#7	BLK	SRC	91%	96%	87%
Patient-Derived	#8	SRMS	SRC	87%	96%	79%
Patient-Derived	#9	ITK	TEC	86%	91%	80%
Patient-Derived	#10	FRK	FRK	84%	78%	91%
All	#1	DDR2	DDR	100%	100%	-
All	#2	TXK	TEC	96%	96%	97%
All	#3	PTK7	PTK7	96%	-	96%
All	#4	LYN	SRC	96%	91%	100%
All	#5	LCK	SRC	95%	91%	99%
All	#6	TEC	TEC	93%	96%	91%
All	#7	BLK	SRC	90%	91%	88%
All	#8	SRMS	SRC	88%	91%	84%
All	#9	FRK	FRK	87%	83%	92%
All	#10	PDGFRA	PDGFR	84%	87%	81%

**Table 2:** UKA and KRSA weighted average percentile rankings.

Cell Line	Rank	Kinase	Family	Weighted Average	KRSA	UKA
PANC1	#1	LCK	SRC	96%	91%	100%
PANC1	#2	LYN	SRC	95%	91%	99%
PANC1	#3	SRC	SRC	92%	91%	92%
PANC1	#4	ABL1	ABL	91%	87%	95%
PANC1	#5	TEC	TEC	90%	100%	80%
PANC1	#6	FYN	SRC	90%	91%	88%
PANC1	#7	BLK	SRC	89%	91%	87%
PANC1	#8	TXK	TEC	89%	100%	77%
PANC1	#9	SRMS	SRC	88%	91%	85%
PANC1	#10	ABL2	ABL	88%	87%	89%
PDCL15	#1	LCK	SRC	98%	96%	100%
PDCL15	#2	LYN	SRC	97%	96%	99%
PDCL15	#3	TEC	TEC	94%	91%	97%
PDCL15	#4	SRC	SRC	94%	96%	92%
PDCL15	#5	FYN	SRC	93%	96%	91%
PDCL15	#6	PDGFRA	PDGFR	90%	87%	93%
PDCL15	#7	FRK	FRK	89%	83%	96%
PDCL15	#8	BLK	SRC	88%	96%	81%
PDCL15	#9	HCK	SRC	86%	96%	77%
PDCL15	#10	TXK	TEC	86%	91%	80%
PDCL5	#1	ROS1	SEV	97%	100%	95%
PDCL5	#2	ALK	ALK	86%	74%	97%
PDCL5	#3	TXK	TEC	83%	65%	100%
PDCL5	#4	LTK	ALK	80%	74%	86%
PDCL5	#5	ITK	TEC	79%	65%	93%
PDCL5	#6	FLT1	VEGFR	78%	91%	65%
PDCL5	#7	EPHB1	EPH	76%	61%	92%
PDCL5	#8	EPHB3	EPH	74%	61%	88%
PDCL5	#9	BTK	TEC	72%	65%	78%
PDCL5	#10	EGFR	EGFR	70%	83%	58%
Patient-Derived	#1	LYN	SRC	96%	96%	96%
Patient-Derived	#2	TXK	TEC	95%	91%	99%
Patient-Derived	#3	TEC	TEC	94%	91%	97%
Patient-Derived	#4	LCK	SRC	92%	96%	88%
Patient-Derived	#5	BLK	SRC	91%	96%	87%
Patient-Derived	#6	SRMS	SRC	87%	96%	79%
Patient-Derived	#7	ITK	TEC	86%	91%	80%
Patient-Derived	#8	FRK	FRK	84%	78%	91%
Patient-Derived	#9	ROS1	SEV	80%	74%	85%
Patient-Derived	#10	HCK	SRC	78%	96%	60%
All	#1	TXK	TEC	96%	96%	97%
All	#2	LYN	SRC	96%	91%	100%
All	#3	LCK	SRC	95%	91%	99%
All	#4	TEC	TEC	93%	96%	91%
All	#5	BLK	SRC	90%	91%	88%
All	#6	SRMS	SRC	88%	91%	84%
All	#7	FRK	FRK	87%	83%	92%
All	#8	PDGFRA	PDGFR	84%	87%	81%
All	#9	SRC	SRC	84%	91%	76%
All	#10	ABL1	ABL	84%	74%	93%

**Table 3:** All pipelines’ (KRSA, UKA, PTM-SEA, and KEA3) average percentile rankings.

Cell Line	Rank	Kinase	Family	Average	KRSA	UKA	PTM-SEA	KEA3
PANC1	#1	DDR2	DDR	97%	96%	-	-	98%
PANC1	#2	TXK	TEC	89%	100%	77%	-	90%
PANC1	#3	SRMS	SRC	86%	91%	85%	-	81%
PANC1	#4	SRC	SRC	82%	91%	92%	55%	91%
PANC1	#5	FYN	SRC	81%	91%	88%	68%	78%
PANC1	#6	MST1R	MET	76%	35%	97%	-	95%
PANC1	#7	INSR	INSR	75%	70%	64%	95%	72%
PANC1	#8	ABL1	ABL	75%	87%	95%	36%	82%
PANC1	#9	FGR	SRC	73%	91%	83%	-	45%
PANC1	#10	KIT	PDGFR	72%	83%	35%	-	97%
PDCL15	#1	DDR2	DDR	99%	100%		-	98%
PDCL15	#2	SRC	SRC	90%	96%	92%	82%	90%
PDCL15	#3	PTK7	PTK7	88%	-	88%	-	
PDCL15	#4	TXK	TEC	88%	91%	80%	-	92%
PDCL15	#5	PDGFRA	PDGFR	86%	87%	93%	68%	97%
PDCL15	#6	MST1R	MET	86%	78%	87%	-	93%
PDCL15	#7	SRMS	SRC	84%	96%	73%	-	82%
PDCL15	#8	KIT	PDGFR	78%	87%	49%	-	97%
PDCL15	#9	INSR	INSR	73%	70%	57%	100%	65%
PDCL15	#10	TEC	TEC	72%	91%	97%	-	28%
PDCL5	#1	PTK7	PTK7	99%	-	99%	-	-
PDCL5	#2	ROS1	SEV	97%	100%	95%	-	-
PDCL5	#3	DDR2	DDR	92%	87%	-	-	97%
PDCL5	#4	TXK	TEC	87%	65%	100%	-	95%
PDCL5	#5	EPHB3	EPH	82%	61%	88%	-	98%
PDCL5	#6	LTK	ALK	80%	74%	86%	-	-
PDCL5	#7	EPHB1	EPH	76%	61%	92%	-	76%
PDCL5	#8	FLT4	VEGFR	76%	91%	43%	-	93%
PDCL5	#9	ITK	TEC	72%	65%	93%	-	57%
PDCL5	#10	FLT1	VEGFR	72%	91%	65%	-	59%
Patient-Derived	#1	PTK7	PTK7	100%	-	100%	-	-
Patient-Derived	#2	DDR2	DDR	99%	100%	-	-	97%
Patient-Derived	#3	TXK	TEC	92%	91%	99%	-	85%
Patient-Derived	#4	SRMS	SRC	84%	96%	79%	-	76%
Patient-Derived	#5	LCK	SRC	80%	96%	88%	60%	78%
Patient-Derived	#6	ROS1	SEV	80%	74%	85%	-	-
Patient-Derived	#7	SRC	SRC	79%	96%	45%	96%	81%
Patient-Derived	#8	EPHB3	EPH	77%	65%	75%	-	93%
Patient-Derived	#9	FLT3	PDGFR	74%	87%	36%	-	99%
Patient-Derived	#10	ITK	TEC	74%	91%	80%	-	50%
All	#1	DDR2	DDR	99%	100%	-	-	97%
All	#2	PTK7	PTK7	96%	-	96%	-	-
All	#3	TXK	TEC	93%	96%	97%	-	85%
All	#4	SRC	SRC	85%	91%	76%	91%	82%
All	#5	SRMS	SRC	84%	91%	84%	-	76%
All	#6	LCK	SRC	81%	91%	99%	55%	78%
All	#7	PDGFRA	PDGFR	75%	87%	81%	36%	96%
All	#8	ROS1	SEV	74%	70%	79%	-	-
All	#9	FLT3	PDGFR	73%	87%	33%	-	99%
All	#10	ITK	TEC	72%	96%	65%	-	56%

**Table 4:** All pipelines’ (KRSA, UKA, PTM-SEA, and KEA3) weighted average percentile rankings.

Cell Line	Rank	Kinase	Family	Weighted Average	KRSA	UKA	PTM-SEA	KEA3
PANC1	#1	SRC	SRC	82%	91%	92%	55%	91%
PANC1	#2	FYN	SRC	81%	91%	88%	68%	78%
PANC1	#3	INSR	INSR	75%	70%	64%	95%	72%
PANC1	#4	ABL1	ABL	75%	87%	95%	36%	82%
PANC1	#5	LCK	SRC	71%	91%	100%	9%	85%
PANC1	#6	PDGFRA	PDGFR	70%	83%	91%	9%	97%
PANC1	#7	TXK	TEC	67%	100%	77%	-	90%
PANC1	#8	RET	RET	67%	26%	77%	77%	87%
PANC1	#9	EPHA2	EPH	65%	30%	59%	91%	81%
PANC1	#10	SRMS	SRC	64%	91%	85%	-	81%
PDCL15	#1	SRC	SRC	90%	96%	92%	82%	90%
PDCL15	#2	PDGFRA	PDGFR	86%	87%	93%	68%	97%
PDCL15	#3	INSR	INSR	73%	70%	57%	100%	65%
PDCL15	#4	LYN	SRC	72%	96%	99%	59%	34%
PDCL15	#5	PDGFRB	PDGFR	71%	87%	65%	91%	41%
PDCL15	#6	LCK	SRC	70%	96%	100%	0%	85%
PDCL15	#7	EPHA2	EPH	69%	74%	28%	91%	82%
PDCL15	#8	TXK	TEC	66%	91%	80%	-	92%
PDCL15	#9	FYN	SRC	66%	96%	91%	0%	76%
PDCL15	#10	MST1R	MET	64%	78%	87%	-	93%
PDCL5	#1	ALK	ALK	70%	74%	97%	63%	45%
PDCL5	#2	ZAP70	SYK	65%	52%	69%	70%	70%
PDCL5	#3	TXK	TEC	65%	65%	100%	-	95%
PDCL5	#4	JAK2	JAK	65%	43%	91%	53%	73%
PDCL5	#5	EGFR	EGFR	62%	83%	58%	67%	42%
PDCL5	#6	KDR	VEGFR	62%	91%	39%	30%	89%
PDCL5	#7	EPHB3	EPH	62%	61%	88%	-	98%
PDCL5	#8	AXL	AXL	59%	57%	28%	97%	54%
PDCL5	#9	CSK	CSK	59%	13%	72%	87%	63%
PDCL5	#10	INSR	INSR	58%	78%	23%	70%	61%
Patient-Derived	#1	LCK	SRC	80%	96%	88%	60%	78%
Patient-Derived	#2	SRC	SRC	79%	96%	45%	96%	81%
Patient-Derived	#3	LYN	SRC	72%	96%	96%	64%	32%
Patient-Derived	#4	PDGFRA	PDGFR	72%	87%	53%	52%	96%
Patient-Derived	#5	TXK	TEC	69%	91%	99%	-	85%
Patient-Derived	#6	INSR	INSR	66%	83%	21%	100%	60%
Patient-Derived	#7	EGFR	EGFR	64%	61%	41%	92%	63%
Patient-Derived	#8	EPHA2	EPH	64%	65%	39%	80%	72%
Patient-Derived	#9	SRMS	SRC	63%	96%	79%	-	76%
Patient-Derived	#10	EPHB3	EPH	58%	65%	75%	-	93%
All	#1	SRC	SRC	85%	91%	76%	91%	82%
All	#2	LCK	SRC	81%	91%	99%	55%	78%
All	#3	PDGFRA	PDGFR	75%	87%	81%	36%	96%
All	#4	LYN	SRC	72%	91%	100%	59%	38%
All	#5	TXK	TEC	70%	96%	97%	-	85%
All	#6	INSR	INSR	68%	78%	32%	100%	62%
All	#7	EPHA2	EPH	64%	57%	43%	86%	72%
All	#8	JAK2	JAK	64%	61%	68%	64%	63%
All	#9	SRMS	SRC	63%	91%	84%	-	76%
All	#10	FYN	SRC	62%	91%	59%	23%	74%

## Supplementary Materials

The following are available online at https://www.mdpi.com/1422-0067/21/22/8679/s1, Table S1: Output data table.

## Abbreviations

PDAC	Pancreatic ductal adenocarcinoma
KRSA	Kinome Random Sampling Analyzer
UKA	Upstream Kinase Analysis
PTM-SEA	Post-Translational Modification Signature Enrichment Analysis
KEA3	Kinase Enrichment Analysis Version 3
Z	Standard score
FC	Fold change
LFC	Log fold change
R2	R-squared statistical measure
HGNC	HUGO Gene Nomenclature Committee
ssGSEA	Single sample Gene Set Enrichment Analysis
PDCL5	Patient-derived pancreatic ductal adenocarcinoma cell line 5
PDCL15	Patient-derived pancreatic ductal adenocarcinoma cell line 15
SNP	Single nucleotide polymorphism
PDX1, IPF1	Pancreatic and duodenal homeobox 1 transcription factor
BSA	Bovine serum albumin
LCK	LCK proto-oncogene, Src family tyrosine kinase
DDR2	Discoidin domain receptor tyrosine kinase 2
LYN	LYN proto-oncogene, Src family tyrosine kinase
SRC	SRC proto-oncogene, non-receptor tyrosine kinase
ABL1	ABL proto-oncogene 1, non-receptor tyrosine kinase
TEC	Tec protein tyrosine kinase
FYN	FYN proto-oncogene, Src family tyrosine kinase
BLK	BLK proto-oncogene, Src family tyrosine kinase
TXK	TXK tyrosine kinase
SRMS	Src-related kinase lacking C-terminal regulatory tyrosine and N-terminal myristylation sites
PDGFRA	Platelet-derived growth factor receptor alpha
FRK	Fyn-related Src family tyrosine kinase
PTK7	Protein tyrosine kinase 7 (inactive)
ROS1	ROS proto-oncogene 1, receptor tyrosine kinase
TNK2	Tyrosine kinase non receptor 2
ALK	ALK receptor tyrosine kinase
LTK	Leukocyte receptor tyrosine kinase
ITK	IL2 inducible T cell kinase
FLT1	Fms-related receptor tyrosine kinase 1
EPHB1	EPH receptor B1
ABL2	ABL proto-oncogene 2, non-receptor tyrosine kinase
HCK	HCK proto-oncogene, Src family tyrosine kinase
EPHB3	EPH receptor B3
BTK	Bruton tyrosine kinase
EGFR	Epidermal growth factor receptor
MST1R	Macrophage stimulating 1 receptor
INSR	Insulin receptor
FGR	FGR proto-oncogene, Src family tyrosine kinase
KIT	KIT proto-oncogene, receptor tyrosine kinase
FLT4	Fms-related receptor tyrosine kinase 4
FLT3	Fms-related receptor tyrosine kinase 3
RET	Ret proto-oncogene
EPHA2	EPH receptor A2
PDGFRB	Platelet-derived growth factor receptor beta
ZAP70	Zeta chain of T cell receptor-associated protein kinase 70
JAK2	Janus kinase 2
KDR	Kinase insert domain receptor
AXL	AXL receptor tyrosine kinase
CSK	C-terminal Src kinase
MET	MET proto-oncogene, receptor tyrosine kinase
SEV	Sevenless
SYK	Spleen-associated tyrosine kinase
VEGFR	Vascular endothelial growth factor receptor
TYRO3	TYRO3 protein tyrosine kinase
EPHB4	EPH receptor B4
PTK6	Protein tyrosine kinase 6
YES1	YES proto-oncogene 1, Src family tyrosine kinase
CSF1R	Colony stimulating factor 1 receptor
FES	FES proto-oncogene, tyrosine kinase
INSRR	Insulin receptor-related receptor
FGFR4	Fibroblast growth factor receptor 4
JAK3	Janus kinase 3
MATK	Megakaryocyte-associated tyrosine kinase
FGFR3	Fibroblast growth factor receptor 3
ERBB3	Erb-b2 receptor tyrosine kinase 3
BMX	BMX nonreceptor tyrosine kinase
IGF1R	Insulin-like growth factor 1 receptor
NTRK1	Neurotrophic receptor tyrosine kinase 1
EPHA4	EPH receptor A4
EPHB2	EPH receptor B2
NTRK3	Neurotrophic receptor tyrosine kinase 3
FER	FER tyrosine kinase
FGFR2	Fibroblast growth factor receptor 2
EPHA1	EPH receptor A1
ERBB4	Erb-b2 receptor tyrosine kinase 4
FGFR1	Fibroblast growth factor receptor 1
DDR1	Discoidin domain receptor tyrosine kinase 1
EPHA5	EPH receptor A5
JAK1	Janus kinase 1
EPHA7	EPH receptor A7
ERBB2	Erb-b2 receptor tyrosine kinase 2
NTRK2	Neurotrophic receptor tyrosine kinase 2
TYK2	Tyrosine kinase 2
PTK2	Protein tyrosine kinase 2
SLTM	SAFB-like transcription modulator
EPHA8	EPH receptor A8
EPHA3	EPH receptor A3
MERTK	MER proto-oncogene, tyrosine kinase
RYK	Receptor-like tyrosine kinase
PTK2B	Protein tyrosine kinase 2 beta
STYK1	Serine/threonine/tyrosine kinase 1
TEK	TEK receptor tyrosine kinase
AATK	Apoptosis-associated tyrosine kinase
MTTP	Microsomal triglyceride transfer protein
TPM3	Tropomyosin 3

## Appendix A

**Table A1:** UKA mean kinase statistic describes direction of differential kinase activity.

Cell Line	Kinase	Mean Kinase Statistic	Direction
PDCL15	BLK	10.30884201	Increased
PANC1	BLK	3.809077325	Increased
PDCL5	BLK	0.963873271	Increased
PDCL15	EGFR	6.92042103	Increased
PANC1	EGFR	2.196020549	Increased
PDCL5	EGFR	0.723998698	Increased
PDCL15	EphA2	6.538112723	Increased
PANC1	EphA2	2.767994333	Increased
PDCL5	EphA2	0.663688934	Increased
PDCL15	FLT4	5.969120078	Increased
PANC1	FLT4	1.888408842	Increased
PDCL5	FLT4	0.754741786	Increased
PDCL15	FRK	10.54460498	Increased
PANC1	FRK	3.517514717	Increased
PDCL5	FRK	0.581814545	Increased
PDCL15	Fyn	11.52215741	Increased
PANC1	Fyn	4.046633984	Increased
PDCL5	Fyn	0.080983021	Increased
PDCL15	InSR	8.923873567	Increased
PANC1	InSR	3.024794143	Increased
PDCL5	InSR	0.508668865	Increased
PDCL15	Lck	12.35321414	Increased
PANC1	Lck	4.06073018	Increased
PDCL5	Lck	0.177602219	Increased
PDCL15	Lyn	11.88473844	Increased
PANC1	Lyn	4.172287974	Increased
PDCL5	Lyn	0.620188468	Increased
PDCL15	PDGFR[alpha]	14.1657858	Increased
PANC1	PDGFR[alpha]	4.47805184	Increased
PDCL5	PDGFR[alpha]	−0.21206998	Decreased
PDCL15	Src	10.51520452	Increased
PANC1	Src	3.487086518	Increased
PDCL5	Src	0.34051653	Increased
PDCL15	TEC	10.09732985	Increased
PANC1	TEC	3.229557798	Increased
PDCL5	TEC	0.732187188	Increased
PDCL15	HCK	10.2005583	Increased
PANC1	HCK	3.13041308	Increased
PDCL5	HCK	0.537561913	Increased
PDCL15	Arg	9.644954083	Increased
PANC1	Arg	3.436290625	Increased
PDCL5	Arg	0.713079917	Increased
PDCL15	DDR1	7.462484096	Increased
PANC1	DDR1	2.528789422	Increased
PDCL5	DDR1	−0.255600871	Decreased
PDCL15	EphA8	9.12751897	Increased
PANC1	EphA8	1.855262187	Increased
PDCL5	EphA8	−0.456838117	Decreased

**Table A2:** Cell line profiles.

Cell Line	Category	Clinicopathological Data of the Patient of Origin	Standard of Care	Mutational Profile	Ref.
PANC1	Commercial	Age: 56; Gender: Male; Ethnicity: Caucasian; Disease: Epithelioid Carcinoma of Ductal Cell Origin	Surgical resection with or without post-surgical adjuvant therapy	*KRAS_G12D; TP53_R273H*	[98]
PDCL15	Patient Derived	Age: 66; Gender: Male; Ethnicity: Caucasian; Disease: Pancreatic Ductal Adenocarcinoma	Surgical resection with or without post-surgical adjuvant therapy	*KRAS_G12D; TP53_WT;*	[7,9,11,108] Data Repo
PDCL5	Patient Derived	Age: 56; Gender: Male; Ethnicity: Caucasian; Disease: Pancreatic Ductal Adenocarcinoma	Surgical resection with or without post-surgical adjuvant therapy	*KRAS_G12V; TP53_G245S*	[7,9,11,108] Data Repo

## Appendix B

**Table A3:** Approved human gene nomenclature.

Cell Line	Kinase	Mean Kinase Statistic	Direction
AATK	apoptosis associated tyrosine kinase	HGNC:21	17q25.3
ABL1	ABL proto-oncogene 1, non-receptor tyrosine kinase	HGNC:76	9q34.12
ABL2	ABL proto-oncogene 2, non-receptor tyrosine kinase	HGNC:77	1q25.2
ALK	ALK receptor tyrosine kinase	HGNC:427	2p23.2-p23.1
AXL	AXL receptor tyrosine kinase	HGNC:905	19q13.2
BLK	BLK proto-oncogene, Src family tyrosine kinase	HGNC:1057	8p23.1
BMX	BMX non-receptor tyrosine kinase	HGNC:1079	Xp22.2
BTK	Bruton tyrosine kinase	HGNC:1133	Xq22.1
CSF1R	colony stimulating factor 1 receptor	HGNC:2433	5q32
CSK	C-terminal Src kinase	HGNC:2444	15q24.1
DDR1	discoidin domain receptor tyrosine kinase 1	HGNC:2730	6p21.33
DDR2	discoidin domain receptor tyrosine kinase 2	HGNC:2731	1q23.3
EGFR	epidermal growth factor receptor	HGNC:3236	7p11.2
EPHA1	EPH receptor A1	HGNC:3385	7q34-q35
EPHA2	EPH receptor A2	HGNC:3386	1p36.13
EPHA3	EPH receptor A3	HGNC:3387	3p11.1
EPHA4	EPH receptor A4	HGNC:3388	2q36.1
EPHA5	EPH receptor A5	HGNC:3389	4q13.1-q13.2
EPHA7	EPH receptor A7	HGNC:3390	6q16.1
EPHA8	EPH receptor A8	HGNC:3391	1p36.12
EPHB1	EPH receptor B1	HGNC:3392	3q22.2
EPHB2	EPH receptor B2	HGNC:3393	1p36.12
EPHB3	EPH receptor B3	HGNC:3394	3q27.1
EPHB4	EPH receptor B4	HGNC:3395	7q22.1
ERBB2	erb-b2 receptor tyrosine kinase 2	HGNC:3430	17q12
ERBB3	erb-b2 receptor tyrosine kinase 3	HGNC:3431	12q13.2
ERBB4	erb-b2 receptor tyrosine kinase 4	HGNC:3432	2q34
FER	FER tyrosine kinase	HGNC:3655	5q21.3
FES	FES proto-oncogene, tyrosine kinase	HGNC:3657	15q26.1
FGFR1	fibroblast growth factor receptor 1	HGNC:3688	8p11.23
FGFR2	fibroblast growth factor receptor 2	HGNC:3689	10q26.13
FGFR3	fibroblast growth factor receptor 3	HGNC:3690	4p16.3
FGFR4	fibroblast growth factor receptor 4	HGNC:3691	5q35.2
FGR	FGR proto-oncogene, Src family tyrosine kinase	HGNC:3697	1p35.3
FLT1	fms related receptor tyrosine kinase 1	HGNC:3763	13q12.3
FLT3	fms related receptor tyrosine kinase 3	HGNC:3765	13q12.2
FLT4	fms related receptor tyrosine kinase 4	HGNC:3767	5q35.3
FRK	fyn related Src family tyrosine kinase	HGNC:3955	6q22.1
FYN	FYN proto-oncogene, Src family tyrosine kinase	HGNC:4037	6q21
HCK	HCK proto-oncogene, Src family tyrosine kinase	HGNC:4840	20q11.21
IGF1R	insulin like growth factor 1 receptor	HGNC:5465	15q26.3
INSR	insulin receptor	HGNC:6091	19p13.2
INSRR	insulin receptor related receptor	HGNC:6093	1q23.1
ITK	IL2 inducible T cell kinase	HGNC:6171	5q33.3
JAK1	Janus kinase 1	HGNC:6190	1p31.3
JAK2	Janus kinase 2	HGNC:6192	9p24.1
JAK3	Janus kinase 3	HGNC:6193	19p13.11
KDR	kinase insert domain receptor	HGNC:6307	4q12
KIT	KIT proto-oncogene, receptor tyrosine kinase	HGNC:6342	4q12
LCK	LCK proto-oncogene, Src family tyrosine kinase	HGNC:6524	1p35.2
LTK	leukocyte receptor tyrosine kinase	HGNC:6721	15q15.1
LYN	LYN proto-oncogene, Src family tyrosine kinase	HGNC:6735	8q12.1
MATK	megakaryocyte-associated tyrosine kinase	HGNC:6906	19p13.3
MERTK	MER proto-oncogene, tyrosine kinase	HGNC:7027	2q13
MET	MET proto-oncogene, receptor tyrosine kinase	HGNC:7029	7q31
MST1R	macrophage stimulating 1 receptor	HGNC:7381	3p21.31
NTRK1	neurotrophic receptor tyrosine kinase 1	HGNC:8031	1q23.1
NTRK2	neurotrophic receptor tyrosine kinase 2	HGNC:8032	9q21.33
NTRK3	neurotrophic receptor tyrosine kinase 3	HGNC:8033	15q25.3
PDGFRA	platelet derived growth factor receptor alpha	HGNC:8803	4q12
PDGFRB	platelet derived growth factor receptor beta	HGNC:8804	5q32
PTK2	protein tyrosine kinase 2	HGNC:9611	8q24.3
PTK2B	protein tyrosine kinase 2 beta	HGNC:9612	8p21.2
PTK6	protein tyrosine kinase 6	HGNC:9617	20q13.33
PTK7	protein tyrosine kinase 7 (inactive)	HGNC:9618	6p21.1
RET	ret proto-oncogene	HGNC:9967	10q11.21
ROS1	ROS proto-oncogene 1, receptor tyrosine kinase	HGNC:10261	6q22.1
RYK	receptor like tyrosine kinase	HGNC:10481	3q22.2
SLTM	SAFB like transcription modulator	HGNC:20709	15q22.1
SRC	SRC proto-oncogene, non-receptor tyrosine kinase	HGNC:11283	20q11.23
SRMS	src-related kinase lacking C-terminal regulatory tyrosine and N-terminal myristylation sites	HGNC:11298	20q13.33
STYK1	serine/threonine/tyrosine kinase 1	HGNC:18889	12p13.2
SYK	spleen associated tyrosine kinase	HGNC:11491	9q22.2
TEC	tec protein tyrosine kinase	HGNC:11719	4p12-p11
TEK	TEK receptor tyrosine kinase	HGNC:11724	9p21.2
TNK2	tyrosine kinase non receptor 2	HGNC:19297	3q29
TXK	TXK tyrosine kinase	HGNC:12434	4p12
TYK2	tyrosine kinase 2	HGNC:12440	19p13.2
TYRO3	TYRO3 protein tyrosine kinase	HGNC:12446	15q15.1
YES1	YES proto-oncogene 1, Src family tyrosine kinase	HGNC:12841	18p11.32
ZAP70	zeta chain of T cell receptor associated protein kinase 70	HGNC:12858	2q11.2

**Table A4:** Nomenclature mapping.

Pipeline	Default	Standardized
KRSA	TEC	TEC
KRSA	DDR	DDR
KRSA	SRC	SRC
KRSA	ABL	ABL
KRSA	PDGFR	PDGFR
KRSA	FRK	FRK
KRSA	JAK	JAK
KRSA	INSR	INSR
KRSA	FGFR	FGFR
KRSA	TRK	TRK
KRSA	ACK	ACK
KRSA	SEV	SEV
KRSA	VEGFR	VEGFR
KRSA	AXL	AXL
KRSA	FAK	FAK
KRSA	MET	MET
KRSA	EPH	EPH
KRSA	RET	RET
KRSA	SYK	SYK
KRSA	RYK	RYK
KRSA	FER	FER
KRSA	ALK	ALK
KRSA	EGFR	EGFR
KRSA	CSK	CSK
UKA	Lck	Lck
UKA	Lyn	Lyn
UKA	TEC	TEC
UKA	FRK	FRK
UKA	Tyro3/Sky	TYRO3
UKA	PDGFR[alpha]	PDGFRa
UKA	Src	Src
UKA	Fyn	Fyn
UKA	Abl	ABL1
UKA	CCK4/PTK7	PTK7
UKA	Ron	MST1R
UKA	CTK	MATK
UKA	Axl	Axl
UKA	Fes	Fes
UKA	BLK	BLK
UKA	TXK	TXK
UKA	Arg	ABL2
UKA	HCK	HCK
UKA	HER3	ERBB3
UKA	Syk	Syk
UKA	Srm	SRMS
UKA	EphA8	EphA8
UKA	ZAP70	ZAP70
UKA	CSK	CSK
UKA	EphB4	EphB4
UKA	Mer	MERTK
UKA	PDGFR[beta]	PDGFRb
UKA	Met	Met
UKA	FAK1	PTK2
UKA	RYK	RYK
UKA	Fgr	Fgr
UKA	Yes	YES1
UKA	InSR	InSR
UKA	Ret	Ret
UKA	DDR1	DDR1
UKA	LTK	LTK
UKA	FGFR2	FGFR2
UKA	Fer	Fer
UKA	Kit	Kit
UKA	EphA5	EphA5
UKA	EphB1	EphB1
UKA	IGF1R	IGF1R
UKA	Ros	ROS1
UKA	FmS/CSFR	CSF1R
UKA	TRKB	NTRK2
UKA	EphA4	EphA4
UKA	JAK2	JAK2
UKA	ALK	ALK
UKA	FGFR3	FGFR3
UKA	Etk/BMX	BMX
UKA	BTK	BTK
UKA	FGFR1	FGFR1
UKA	TRKC	NTRK3
UKA	EphB3	EphB3
UKA	EphA2	EphA2
UKA	ITK	ITK
UKA	Lmr1	AATK
UKA	EphA1	EphA1
UKA	KDR	KDR
UKA	FGFR4	FGFR4
UKA	FLT3	FLT3
UKA	FAK2	PTK2B
UKA	JAK3	JAK3
UKA	HER2	ERBB2
UKA	IRR	INSRR
UKA	TRKA	NTRK1
UKA	JAK1~b	JAK1
UKA	HER4	ERBB4
UKA	Tyk2	Tyk2
UKA	EphA3	EphA3
UKA	FLT4	FLT4
UKA	Brk	PTK6
UKA	EphA7	EphA7
UKA	EphB2	EphB2
UKA	EGFR	EGFR
UKA	FLT1	FLT1
PTM-SEA	ZAP70	ZAP70
PTM-SEA	VEGFR2/KDR	KDR
PTM-SEA	TrkA/NTRK1	NTRK1
PTM-SEA	Syk/SYK	SYK
PTM-SEA	Src/SRC	SRC
PTM-SEA	Ret/RET	RET
PTM-SEA	PDGFRB	PDGFRB
PTM-SEA	PDGFRA	PDGFRA
PTM-SEA	MKK4/MAP2K4	MAP2K4
PTM-SEA	Met/MET	MET
PTM-SEA	Mer/MERTK	MERTK
PTM-SEA	MEK1/MAP2K1	MAP2K1
PTM-SEA	LYN	LYN
PTM-SEA	Lck/LCK	LCK
PTM-SEA	JAK3	JAK3
PTM-SEA	JAK2	JAK2
PTM-SEA	INSR	INSR
PTM-SEA	IGF1R	IGF1R
PTM-SEA	HER2/ERBB2	ERBB2
PTM-SEA	Fyn/FYN	FYN
PTM-SEA	Fer/FER	FER
PTM-SEA	Etk/BMX	BMX
PTM-SEA	EphA2/EPHA2	EPHA2
PTM-SEA	EGFR	EGFR
PTM-SEA	CSK	CSK
PTM-SEA	Chk1/CHEK1	CHEK1
PTM-SEA	AXL	AXL
PTM-SEA	ALK	ALK
PTM-SEA	Abl/ABL1	ABL1
KEA3	NTRK1	NTRK1
KEA3	FLT3	FLT3
KEA3	DDR2	DDR2
KEA3	KIT	KIT
KEA3	PDGFRA	PDGFRA
KEA3	MATK	MATK
KEA3	EPHB3	EPHB3
KEA3	MST1R	MST1R
KEA3	FES	FES
KEA3	FLT4	FLT4
KEA3	SRC	SRC
KEA3	TXK	TXK
KEA3	NTRK3	NTRK3
KEA3	KDR	KDR
KEA3	RET	RET
KEA3	LCK	LCK
KEA3	ABL1	ABL1
KEA3	EPHA2	EPHA2
KEA3	SRMS	SRMS
KEA3	EPHB2	EPHB2
KEA3	FYN	FYN
KEA3	EGFR	EGFR
KEA3	FLT1	FLT1
KEA3	FER	FER
KEA3	INSR	INSR
KEA3	FGFR4	FGFR4
KEA3	ITK	ITK
KEA3	EPHB1	EPHB1
KEA3	CSF1R	CSF1R
KEA3	PTK6	PTK6
KEA3	CSK	CSK
KEA3	ERBB2	ERBB2
KEA3	NTRK2	NTRK2
KEA3	TYRO3	TYRO3
KEA3	BTK	BTK
KEA3	JAK2	JAK2
KEA3	SYK	SYK
KEA3	LYN	LYN
KEA3	FGFR3	FGFR3
KEA3	PTK2	PTK2
KEA3	FGR	FGR
KEA3	ERBB4	ERBB4
KEA3	YES1	YES1
KEA3	ZAP70	ZAP70
KEA3	JAK3	JAK3
KEA3	MET	MET
KEA3	IGF1R	IGF1R
KEA3	TEC	TEC
KEA3	AXL	AXL
KEA3	ALK	ALK
KEA3	PTK2B	PTK2B
KEA3	PDGFRB	PDGFRB
KEA3	STYK1	STYK1
KEA3	MERTK	MERTK
KEA3	BMX	BMX
KEA3	EPHA3	EPHA3
KEA3	ABL2	ABL2
KEA3	FGFR1	FGFR1
KEA3	EPHA4	EPHA4
KEA3	TYK2	TYK2
KEA3	FRK	FRK
KEA3	FGFR2	FGFR2
KEA3	TNK2	TNK2
KEA3	JAK1	JAK1
KEA3	DDR1	DDR1
KEA3	BLK	BLK
KEA3	HCK	HCK
KEA3	EPHA8	EPHA8
KEA3	TEK	TEK

Figure 2, Figure 3 and Figure 4**, expanded figure legend.** Default outputs from upstream kinase identification pipelines for PDAC cell lines compared to patient-derived wild-type pancreatic tissue. (**A**) The Kinome Random Sampling Analyzer (KRSA) pipeline identifies differentially active upstream kinases according to mean standard score (Z) values on the *x* axis, with kinase family on the *y* axis, and color gradation representing absolute mean standard score. Full red represents an absolute mean standard score above 3 and full white represents an absolute mean standard score below 1. Absolute mean standard scores for each kinase family are calculated by averaging the absolute standard score values of nine standard scores, each derived from input lists consisting of peptide sequences with log-fold change (LFC) in phosphorylation above 0.2, 0.3, or 0.4 in one of our three biological replicates. Each plotted box represents values within and including the first and third percentiles (the 25th and 75th percentiles). Upper or lower whiskers extend from the box to the largest or smallest kinase family standard score that is less than or equal to 150% of the distance between the first and third percentiles, respectively. Outliers defined as greater than 150% of the distance between the first and third percentiles are plotted individually. Dashed lines delineate positive values above 1.5, 1.7, and 2.0 or negative values below −1.5, −1.7, and −2.0. Kinase families appear in descending order of standard score with the most significant differential kinase family activity appearing at the top and bottom of the list. Any zero-value standard score represents a kinase family having identical mean log-fold change values between the phosphorylation levels of representative peptide sequences measured in the PDAC cell line group and the phosphorylation levels of those same representative peptide sequences when measured in the control wild-type patient sample group. (**B**) The Post-Translational Modification Signature Enrichment Analysis (PTM-SEA) pipeline identifies differentially active upstream kinases according to negative decadic logarithms of the adjusted probability values. These values allow the most significant differentially active kinases to be listed from top to bottom in order of descending significance. Adjusted probability values less than 0.05 are, therefore, represented by *x* axis values above 1.30. Adjusted probability values correcting for multiple comparisons use the Bonferroni correction method on comparisons between the phosphorylated peptide sequences of the experimental PDAC cell line group and the phosphorylated peptide sequences of the control wild-type patient group in order to determine the extent to which these differences may be attributed to each individual kinase listed on the left. (**C**) The Kinase Enrichment Analysis Version 3 (KEA3) pipeline also identifies differentially activated upstream kinases according to negative decadic logarithms of the average adjusted probability values and allows the most significant differentially active kinases to be listed from top to bottom in order of descending significance. Adjusted probability values less than 0.05 are, therefore, represented by *x* axis values above 1.30. Adjusted probability values less than 0.01 are represented by *x* axis values above 2. Adjusted probability values correcting for multiple comparisons use the false discovery rate (FDR) correction method to compare the genes containing phosphorylated peptide sequences of the experimental PDAC cell line group with the genes containing phosphorylated peptide sequences of the control wild-type patient group in order to determine the extent to which these differences may be attributed to each individual kinase listed on the left. (**D**) The Upstream Kinase Analysis (UKA) pipeline identifies differentially activated upstream kinases according to the normalized kinase statistic for change in phosphorylation between the peptide sequences of the experimental PDAC cell line group and the control wild-type patient group. This normalized kinase statistic is calculated by subtracting the mean phosphorylation levels of the peptide sequences associated with a given kinase in the control group from the mean phosphorylation levels of the peptide sequences associated with that same kinase in the experimental group and dividing the resulting value by the standard deviation. A quotient above 0 indicates increased activity of a kinase from the experimental group. A quotient below 0 indicates decreased activity of a kinase from the experimental group. Each graphed point represents the normalized kinase statistic when the UKA algorithm uses only one of the pipeline’s available databases. The diameter of a circle increases with the size of the peptide sequences associated with the corresponding kinases in the corresponding database. Color gradation represents specificity scores with full red representing values of two and full black representing specificity scores of zero. Specificity scores are informed by the quotient produced with a dividend corresponding to the number of times that a permuted normalized kinase statistic greatly exceeded the nonpermuted normalized kinase statistic and a divisor corresponding to the number of random permutations, or the quotient produced by the number 1 as a dividend and the number of random permutations as the divisor. Specificity scores represent the negative decadic logarithm of whichever quotient is larger. Individual kinases are listed along the *y* axis from top to bottom in descending order of median “final score.” UKA calculates the “final score” as the summation of the significance score and specificity score of each individual kinase. (**E**) Comparison of upstream kinase activity as determined by KRSA, PTM-SEA, KEA3, and UKA, each listed on the *y* axis. Kinase family names appear along the top of the *x* axis. Individual kinase family members appear along the bottom of the *x* axis. The comparison includes only kinases identified as one of the top 10 most differentially active upstream kinases by any one of the pipelines. Kinases with identical scores were arbitrarily assigned sequential ranks. However, if two or more kinases tied for the last (10th) position in this list, then the list was extended so as not to arbitrarily exclude an equivalently ranked kinase. The diameter of each black circle corresponds with the percentile in which the labeled kinase appears in the labeled pipeline. The kinase family scores of the KRSA pipeline are repeated for each individual kinase. A white circle demonstrates not only absence from a given pipeline’s top 10 list, but also demonstrates absence from a given pipeline’s unfiltered exhaustive output.
